# A pH‐Sensitive Nanosized Covalent–Organic Polymer for Enhanced Tumor Photodynamic Immunotherapy by Hypoxia Relief and STAT3 Inhibition

**DOI:** 10.1002/advs.202504860

**Published:** 2025-05-14

**Authors:** Lei Lei, Wenbin Dai, Jinchao Zhao, Angfeng Jiang, Haisheng Peng, Qiao Jin, Xiaojing Li, Zhe Tang

**Affiliations:** ^1^ Department of General Surgery The Fourth Affiliated Hospital International Institutes of Medicine Zhejiang University School of Medicine Yiwu 322000 China; ^2^ MOE Key Laboratory of Macromolecular Synthesis and Functionalization Department of Polymer Science and Engineering Zhejiang University Hangzhou 310058 China; ^3^ Department of Pharmacology Medical College of Shaoxing University Shaoxing 312099 China; ^4^ Department of Gynecology The Second Affiliated Hospital School of Medicine Zhejiang University Hangzhou 310058 China; ^5^ Department of Surgery The Second Affiliated Hospital School of Medicine Zhejiang University Hangzhou 310058 China

**Keywords:** covalent–organic polymer, hypoxia relief, immunotherapy, nanomedicine, photodynamic therapy

## Abstract

Photodynamic therapy (PDT) is a promising cancer therapy modality by generating reactive oxygen species (ROS) and triggering immunogenic cell death. However, the therapeutic effect of PDT is strongly limited by tumor hypoxia and immunosuppressive landscape. Herein, a pH‐sensitive nanosized covalent–organic polymer (COP), composed of the photosensitizer porphyrin and pyruvate kinase inhibitor vitamin K3 (VK3), is designed to overcome these issues. The signal transducer and activator of transcription 3 (STAT3) inhibitor WP1066 is further encapsulated into COPs to form a WP1066‐loaded COP (TVW). As an inhibitor of pyruvate kinase, VK3 can reduce intracellular oxygen consumption by inhibiting the glycolytic pathway, leading to the alleviation of the tumor hypoxic microenvironment. The relief of tumor hypoxia by VK3 enhances photodynamic cytotoxicity by generating more ROS. Meanwhile, STAT3 acts as a major regulator of PD‐L1, a key inhibitor that promotes immune escape. WP1066 effectively inhibits the expression of STAT3 and reduces PD‐L1 expression, thereby significantly inhibiting tumor immune escape and enhancing antitumor efficacy in a synergistic manner. The antitumor capacity of photodynamic immunotherapy is extensively investigated in a murine subcutaneous hepatocellular carcinoma model. This photo‐immunotherapy may provide an effective combination regimen for the efficient treatment of solid tumors such as hepatocellular carcinoma.

## Graphical abstract

The nanosized covalent organic polymer composed of photosensitizer porphyrin, pyruvate kinase inhibitor vitamin K3, and STAT3 inhibitor WP1066 is prepared for enhanced photodynamic immunotherapy by simultaneous hypoxia relief and STAT3 inhibition.

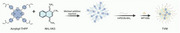

## 1 Introduction

Photodynamic therapy (PDT) is an effective cancer treatment modality with minimal invasion and low side effects. PDT is a form of cancer treatment that involves the conversion of oxygen into highly cytotoxic reactive oxygen species (ROS), which is achieved through the use of photosensitizers (PSs) by light activation at specific wavelengths.^[^ ^1^, ^2^ ^]^ However, the efficacy of most PDT systems is highly contingent upon the concentration of intertumoral oxygen. The quantity of oxygen that can be converted to ROS is a pivotal factor influencing the efficacy of PDT.^[^ ^3^, ^4^ ^]^ At the same time, some studies have also demonstrated that tumor development is accompanied by various mechanisms that can induce a hypoxic tumor microenvironment.^[^ ^5^ ^]^ To illustrate, the characteristics of rapid metabolism and elevated oxygen consumption in tumor cells can induce abnormalities in vascular endothelial structure and function, thereby disrupting oxygen delivery and resulting in a reduction in oxygen supply within the intratumor area, which in turn gives rise to a chronic hypoxic microenvironment.^[^ ^6^, ^7^, ^8^ ^]^ In addition, the consumption of oxygen by PDT also results in a reduction in the effective oxygen content available for conversion to ROS, thereby severely limiting the efficacy of PDT.^[^ ^9^, ^10^, ^11^ ^]^ To address the problem of insufficient oxygen in the tumor microenvironment, many studies have proposed to modulate the hypoxic tumor microenvironment by increasing oxygen supply or reducing oxygen consumption.^[^ ^12^, ^13^, ^14^, ^15^ ^]^

PDT can not only directly eradicate tumor cells via the generation of ROS, but also effectively elicits immunogenic cell death (ICD) in tumor cells.^[^ ^16^, ^17^, ^18^ ^]^ ROS generated by PDT may induce oxidative stress within the endoplasmic reticulum (ER), which could potentially result in the activation of the ICD pathway in tumor cells.^[^ ^19^, ^20^ ^]^ Furthermore, the death of tumor cells may facilitate the generation of novel antigenic epitopes, in addition to the release of damage‐associated molecular patterns (DAMPs), such as calreticulin (CRT) and high mobility group box‐1 (HMGB‐1).^[^ ^21^, ^22^, ^23^ ^]^ The released DAMPs can interact with antigen‐presenting cells (e.g., dendritic cells [DCs]), which recognize and engulf dead cell antigens. These antigens are then presented to T cells, which subsequently activate adaptive immune responses. However, due to the immunosuppressive environment and the low immunogenicity of tumor cells, PDT alone is not sufficient for achieving a robust and sustained antitumor effect in cancer immunotherapy programs.^[^ ^24^, ^25^ ^]^ Consequently, various strategies have been devised to enhance the immune response.^[^ ^26^, ^27^ ^]^

Since photodynamic therapy can cause direct killing of tumor cells and ICD, photodynamic immunotherapy has become a noteworthy antitumor tool.^[^ ^28^, ^29^ ^]^ Among the existing tumor immunotherapies, the inhibition of the PD‐1/PD‐L1 axis has become a principal therapeutic strategy in cancer immunotherapy. PD‐L1 can be up‐regulated in tumor cells during tumor growth, inhibiting T‐cell proliferation and activation, putting T‐cells in an inactivated state and ultimately inducing immune escape. The regulation of PD‐L1 is a complex process involving the participation of multiple factors. Among these, the signal transducer and activator of transcription 3 (STAT3) is a pivotal transcription factor for cell proliferation and tumorigenesis. Activation of STAT3 has been demonstrated to upregulate the transcription of numerous repressor genes, including PD‐L1. Consequently, the inhibition of STAT3 expression can further inhibit the binding of PD‐1 to PD‐L1, thereby preventing tumor immune escape. However immunological drugs used alone are associated with a range of adverse effects, including rash, diarrhea, and pneumonia. Therefore, there is an urgent need to develop effective carriers that can efficiently combine photosensitizers and immunological drugs to achieve tumor photodynamic immunotherapy.^[^ ^30^, ^31^ ^]^

The covalent–organic polymers (COPs) have emerged as a promising class of materials in recent studies.^[^ ^32^, ^33^ ^]^ COPs are framework materials in which organic building blocks are linked by covalent bonds to form periodic, well‐defined structures. The richness of their constituent elements, the flexibility of their structural designability, their large surface area, and their excellent physicochemical properties, such as controllability, tunability, and stability, make them an ideal choice for use as an anticancer drug delivery system with high specificity and efficacy.

**Scheme 1:**
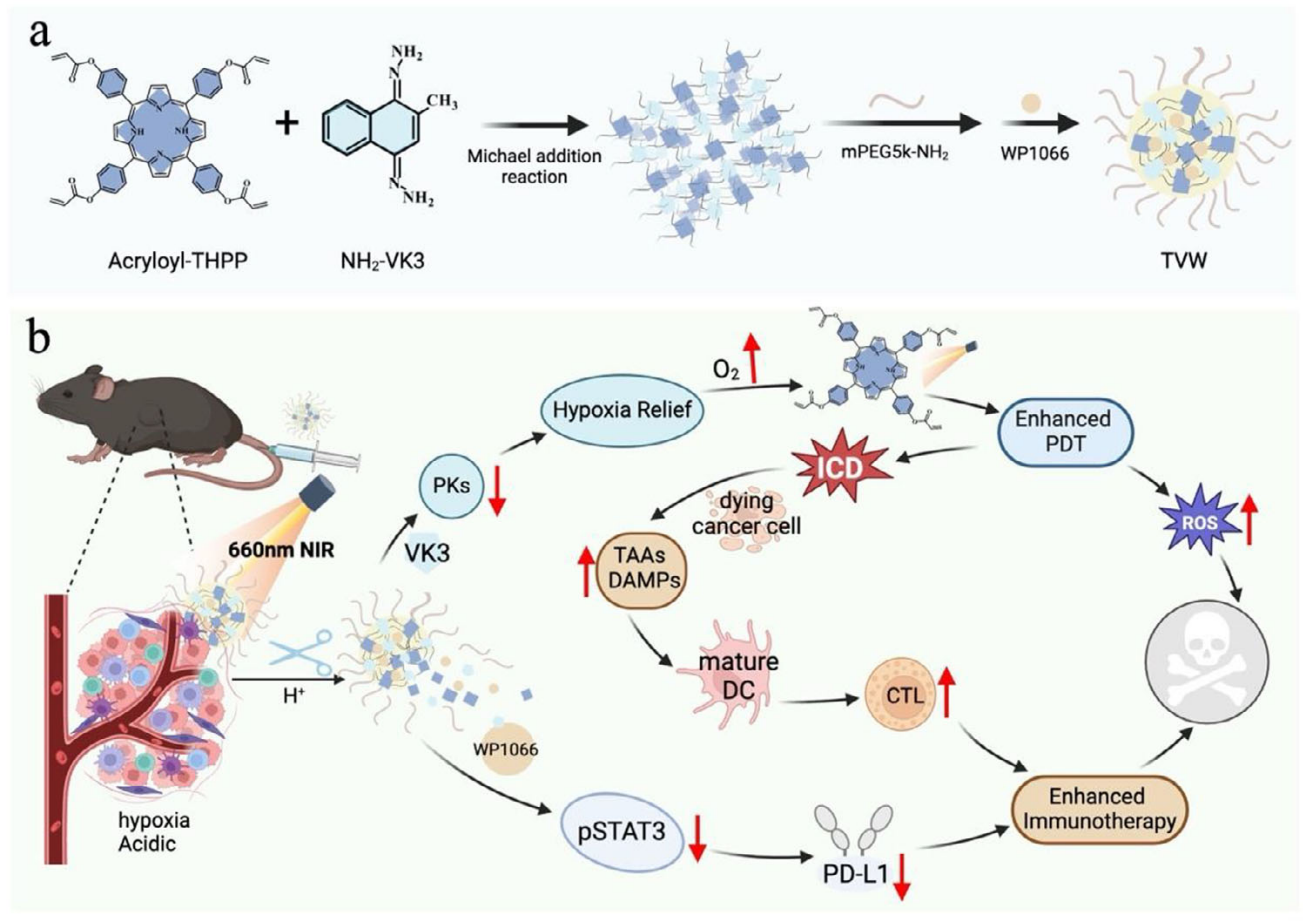
Schematic illustration of the preparation and potential antitumor mechanism of TVW. a) The preparation of TVW. b) The mechanism of TVW enhanced photodynamic immunotherapy by relieving tumor hypoxia and immunosuppressive landscape. Created by biorender.

To enhance the therapeutic efficacy of photodynamic immunotherapy, it is critical to relieve tumor hypoxia and immunosuppressive landscape. In this study, a pH‐sensitive nanosized COP composed of photosensitizer porphyrin and pyruvate kinase (PK) inhibitor vitamin K3 (VK3) was designed to address these challenges (**Scheme** **1**). The STAT3 inhibitor WP1066 was further encapsulated to form WP1066‐loaded COPs (denoted as TVW). As an inhibitor of pyruvate kinase, VK3 could relieve tumor hypoxic microenvironment by reducing oxygen consumption. Thus, the presence of VK3 enables PDT to utilize more oxygen to generate cytotoxic ROS, thereby exerting a more pronounced cytotoxic effect on tumor cells. Furthermore, the STAT3 inhibitor WP1066 could inhibit the phosphorylation of STAT3, reduce the expression of PD‐L1, impede the PD‐1/PD‐L1 binding, restore cytotoxic T‐lymphocyte (CTL) function, and regulate the immunosuppressive microenvironment.^[^ ^34^, ^35^ ^]^ The therapeutic efficacy of TVW‐based tumor photodynamic immunotherapy was expected to be improved by simultaneous modulation of tumor hypoxia and immunosuppressive landscape.

## 2 Results and Discussion

### 2.1 Preparation and Characterization of TT, TV, and TVW

The acid‐cleavable nanoscale COPs composed of photosensitizer porphyrin and pyruvate kinase inhibitor VK3 were prepared by the polymerization of acryloyl‐THPP and NH_2_‐VK3 via a Michael addition reaction (Figures S1–S3, Supporting Information). As evidenced by the ^1^H NMR, a chemical shift at δ 6.75–7.5 ppm was indicative of the successful modification of the amino groups, confirming the successful synthesis of NH_2_‐VK3 (Figures S4 and S5, Supporting Information). Meanwhile, a chemical shift at δ 6.04–6.80 ppm was observed in Figure S6 (Supporting Information), indicating the successful introduction of acryloyl groups to THPP. The successful synthesis of acryloyl‐THPP was also confirmed by its ^13^C NMR spectrum (Figure S7, Supporting Information).

**Figure 1:**
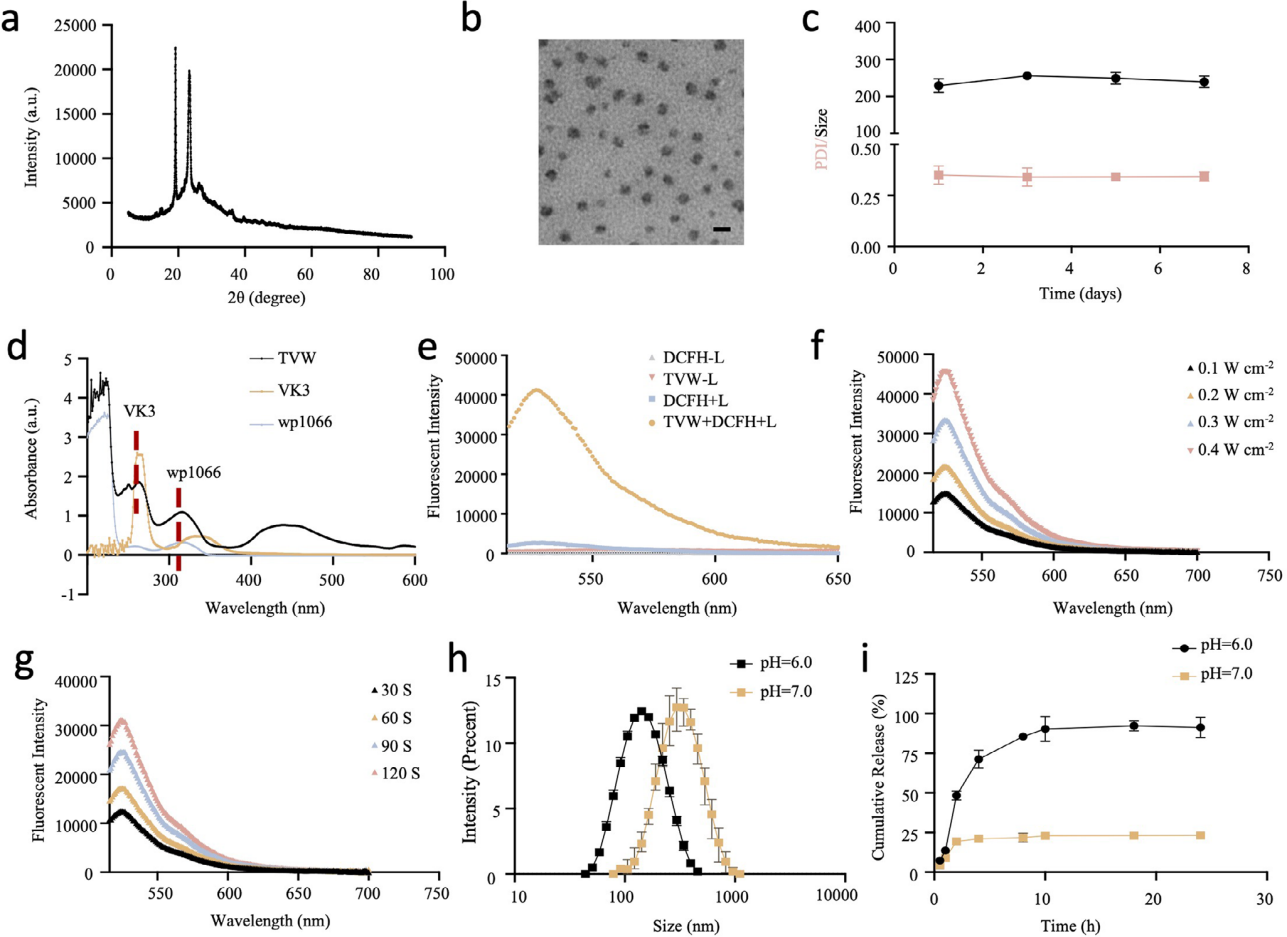
Characterization of COPs. a) XRD result of TVW. b) TEM image of TVW. Scale bar: 200 nm. c) Hydrodynamic diameters (upper) and PDI changes (lower) of TVW upon incubating in PBS for 7 d. d) UV–vis spectra of TT and TVW. e) The generation of ROS was detected by fluorescent spectrum after TVW and DCFH‐DA was irradiated by 660 nm laser. DCFH‐DA was used as ROS fluorescent probe. f) The generation of ROS was detected by fluorescent spectrum after TVW was irradiated by 660 nm laser with different light intensity (0.1, 0.2, 0.3, 0.4 W cm^−2^). g) The generation of ROS was detected by fluorescent spectrum after TVW was irradiated by 660 nm laser with different irradiation time (30, 60, 90, 120 s). h) The hydrodynamic diameters of TVW at pH 7.0 and 6.0. i) The accumulative release of WP1066 from TVW at pH 6.0 and pH 7.0.

The physical structure and optical properties of COPs were further investigated. The crystal phase of TVW was confirmed by X‐ray diffraction (XRD, **Figure** **1**a). The diffraction peaks were sharp and intense, indicating the highly crystalline nature of TVW. The morphology of TVW was subsequently observed using transmission electron microscopy (TEM), which revealed that all the nanoparticles exhibited a spherical morphology with an average diameter of ≈100 nm (Figure 1b). In order to guarantee the effective circulation of nanoparticles in systemic venous blood, it is of the utmost importance that their superior particle stability is ensured. Dynamic light scattering (DLS) was used to evaluate the stability of TVW. The particle size and polydispersity index (PDI) of TVW did not show significant change during the observation period, indicating excellent stability of TVW (Figure 1c). TT and TV also maintained excellent stability over 7 d detected by DLS (Figure S8, Supporting Information). These findings supported the feasibility of subsequent animal experiments. We analyzed the XPS spectra to determine the elemental composition of TVW. As shown in Figure S9 (Supporting Information), TVW contained C, N, O, and Br. Br is a unique element of WP1066. The presence of Br in TVW confirmed the successful loading of WP1066 in TVW. The surface charge of TVW at different pH was then investigated by measuring the zeta potential. As illustrated in Figure S10 (Supporting Information), the zeta potentials of TVW were ≈−6 and −4 mV at pH 7.0 and pH 6.0, respectively. Furthermore, the characteristic peaks of VK3 and WP1066 at 230 and 317 nm were observed in the ultraviolet–visible (UV–vis) spectrum of TVW (Figure 1d), thereby confirming the successful loading of VK3 and WP1066 in TVW. FTIR spectra further confirmed the payload of wp1066 (Figure S11, Supporting Information). In addition, by analyzing the absorbance of photosensitizer THPP at its characteristic peaks at different concentrations, a standard calibration curve can be obtained for the calculation of drug concentration in TVW (Figure S12, Supporting Information).

In this study, THPP acted as a photosensitizer that could convert oxygen to ROS under 660 nm laser light, resulting in the killing of tumor cells. The photodynamic effect of TVW was firstly investigated. The 2′,7′‐dichlorodihydrofluorescein diacetate (DCFH‐DA) fluorescent probe can be enzymatically hydrolyzed to DCFH and then oxidized to fluorescent 2′,7′‐dichlorofluorescein (DCF) by ROS after entering the cell. Therefore, DCFH‐DA was used as a ROS fluorescent probe in this study to assess the ability of THPP to generate ROS after laser irradiation. As shown in Figure 1e, DCFH did not show obvious fluorescence of ROS probe either in the dark or under light exposure. In the TVW group, the fluorescence of ROS probe was also not detected without light irradiation. However, after irradiating TVW with a 660 nm laser, the characteristic fluorescence of DCF was observed at a wavelength of 495 nm, which indicated the generation of ROS in the TVW group after laser irradiation. Subsequently, the dependence of the photodynamic effect of TVW on irradiation time and light intensity was investigated. As shown in Figure 1f,g, the fluorescence intensity increased significantly with the increase of light intensity and irradiation time, which indicated that ROS production can be well regulated by light intensity and irradiation time. In addition, we analyzed if the encapsulation of THPP in COPs can influence ROS generation. As shown in Figure S13 (Supporting Information), neither the incorporation of THPP in COPs nor the loading of WP1066 in COPs had a significant effect on the ROS generation ability of THPP. Due to the presence of acid‐cleavable backbone in TVW, TVW may degrade in the acidic tumor microenvironment, triggering the dissociation of TVW and further releasing VK3 and WP1066. The intensity‐averaged hydrodynamic diameter of TVW was ≈190 nm at pH 7.0 measured by DLS, while the diameter of TVW was increased to 345 nm at pH 6.0 (Figure 1h). To evaluate the pH‐triggered drug release characteristics, we investigated the cumulative release of WP1066 from TVW in aqueous solutions at pH 6.0 and 7.0 (Figure 1i). The pH‐responsive release behavior as well as accelerated drug release from TVW was observed at pH 6.0. More than 80% WP1066 was released from TVW after 24 h incubation at pH 6.0. However, only about 20% WP1066 was released from TVW after 24 h incubation at pH 7.0. The acid‐responsive drug release of COPs was very beneficial to reduce the adverse effects in cancer therapy.

### 2.2 TVW‐Triggered Photodynamic Therapy In Vitro

**Figure 2:**
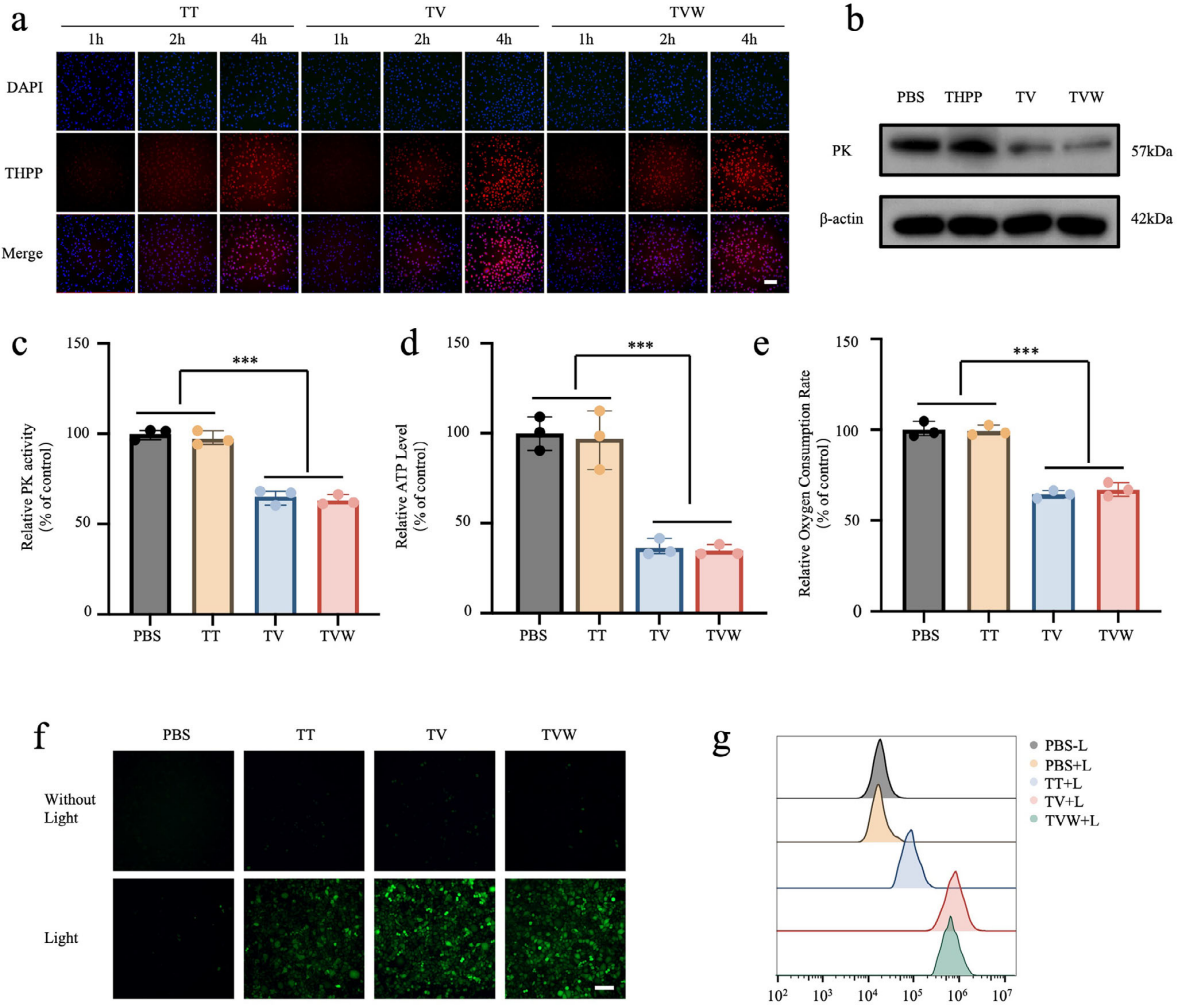
Photodynamic effect of TVW in vitro. a) Fluorescence micrographs of Hepa1‐6 cells after incubation with TT, TV, and TVW for different time intervals. DAPI (Ex: 358 nm, Em: 461 nm), THPP (Ex: 405 nm, Em: 650 nm). Scale bar: 100 µm. b) The detection of PK level by WB assay after Hepa1‐6 cells were treated with PBS, TT, TV, and TVW. c) Relative PK level in Hepa1‐6 cells after treatment with PBS, TT, TV, and TVW. d) Relative ATP level in Hepa1‐6 cells after treatment with PBS, TT, TV, and TVW. e) The oxygen consumption rate in Hepa1‐6 cells after treatment with PBS, TT, TV, and TVW. f) The production of ROS detected by DCFH‐DA in Hepa1‐6 cells. The cells were treated with PBS, TT, TV, and TVW. Ex: 495 nm, Em: 519 nm. Scale bar: 100 µm. g) The production of ROS detected by flow cytometry in Hepa1‐6 cells. The cells were treated PBS‐L, PBS+L, TT+L, TV+L, TVW+L. “+L” means “with light irradiation.” Data were expressed as means ± SD. *p < 0.05, **p < 0.01, ***p < 0.001.

The prerequisite for nanomedicines to be effective in vivo is that they can be internalized by tumor cells. Since porphyrin in COPs can be used as a fluorescent probe to study the internalization of COPs, we investigated the uptake of nanomedicines by Hepa1‐6 cells using fluorescence microscopy. As shown in **Figure** **2**a, Hepa1‐6 cells were incubated with TT, TV, and TVW, and the cellular uptake of the drugs was observed at three‐time points: 1, 2, and 4 h, respectively. The red fluorescence emitted from porphyrin could be observed within the cells, indicating that all three COP nanoparticles could be efficiently internalized by Hepa1‐6 cells. Furthermore, it was found that the intensity of intracellular red fluorescence exhibited a time dependence, with the prolongation of the incubation time increasing the fluorescence signal. The uptake behavior of Hepa1‐6 cells was also analyzed by flow cytometry at 1, 2, and 4 h, respectively. A quantitative analysis was performed to verify the efficient internalization of TVW by Hepa1‐6 cells and to identify an increase in drug uptake over time (Figure S14, Supporting Information). The findings were consistent with the microscopic observations of cellular uptake, which demonstrated the efficient uptake of COP nanoparticles by tumor cells.

It has also been demonstrated that VK3 is an efficacious inhibitor of PK, which impedes energy metabolism in tumor cells by affecting the glycolytic pathway.^[^ ^36^, ^37^ ^]^ PK is the final key enzyme involved in glycolysis. Therefore, the introduction of VK3 in TVW may reduce intracellular oxygen consumption by inhibiting glycolysis. Following incubation of TT, TV, and TVW for 24 h, PK expression was quantified by western blotting (WB). As shown in Figure 2b and Figure S15 (Supporting Information). TT treatment did not influence the expression of PK. However, VK3‐containing TV and TVW resulted in a decrease of PK level. Furthermore, the intracellular PK level was assessed using a PK activity kit (Figure 2c). As expected, VK3 could result in 62.9% reduction of PK activity, consistent with the WB results. Given that tumor cells exhibit the Warburg effect during normal metabolic processes, tumor cells depend on glycolysis as a primary source of energy. Consequently, when glycolysis is inhibited, the capacity of tumor cells to produce ATP via this pathway is substantially reduced. Therefore, the degree of glycolysis inhibition can be quantified by measuring intracellular ATP levels. ATP levels were assessed using an ATP assay kit to ascertain the extent of glycolysis inhibition in the various treatment groups. As shown in Figure 2d, TT had no impact on intracellular ATP production. However, Hepa1‐6 cells incubated with TV and TVW demonstrated a significant reduction in intracellular ATP levels, indicating that VK3 effectively blocked energy supply. Subsequently, the oxygen consumption rate (OCR) of Hepa1‐6 cells was quantified following various treatments utilizing the OCR assay kit (Figure 2e). In accordance with the aforementioned results, the oxygen consumption rate of Hepa1‐6 cells was reduced to 66.4% after incubated with TV and TVW. In conclusion, as a glycolysis inhibitor, VK3 can reduce intracellular oxygen consumption by inhibiting the activity of PK, thus increasing the intracellular accumulation of oxygen. Since oxygen is the “combustion agent” of PDT, the incorporation of VK3 into COPs was expected to effectively alleviate tumor hypoxia, which was anticipated to enhance the therapeutic efficacy of PDT.

To investigate the improvement of photodynamic effect of COPs after the introduction of VK3 in the framework of photosensitizers, the production of intracellular ROS in Hepa1‐6 cells after incubation with TT, TV, and TVW was quantified using DCFH‐DA as a ROS probe. As illustrated in Figure 2f, the “without Light” and “Light” groups were established, respectively. The green fluorescence could not be observed in any of the “without Light” groups, indicating that the photosensitizer could only be excited under laser irradiation. Following irradiation with a 660 nm laser, the TT+L group exhibited a relatively low level of green fluorescence. However, the cells treated with TV+L and TVW+L exhibited enhanced green fluorescence, indicating that the intracellular ROS levels could be effectively elevated in the presence of VK3. Therefore, VK3 may enhance the photodynamic effect of TT. Meanwhile, flow cytometry was used to quantify the intracellular fluorescence of ROS probe after different treatments, further verifying that VK3 could enhance the ability of COP nanoparticles to produce ROS, paving the way for the generation of a more intense photodynamic effect (Figure 2g and Figure S16, Supporting Information).

### 2.3 TVW‐Triggered ICD In Vitro

**Figure 3:**
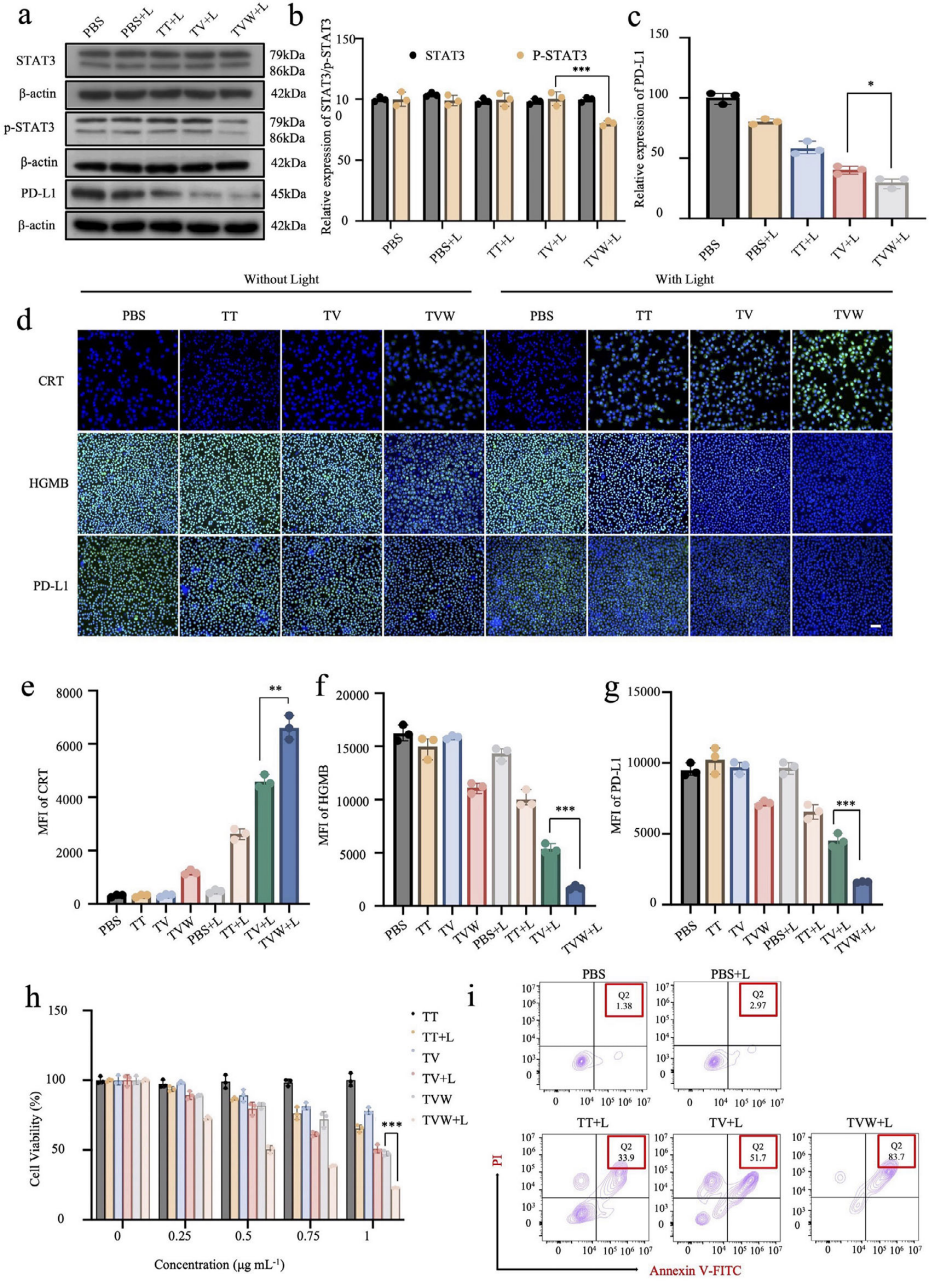
Immune effect of TVW in vitro. a) The detection of STAT3, pSTAT3, and PD‐L1 level by WB assay after Hepa1‐6 cells were incubated with PBS, TT, TV, and TVW. b) Quantification analysis of relative levels of STAT3 and pSTAT3 in Hepa1‐6 cells treated with PBS, TT, TV, TVW, PBS+L, TT+L, TV+L, and TVW+L. “+L” means “with light irradiation.” c) Quantification analysis of relative levels of PD‐L1 in Hepa1‐6 cells incubated with PBS, TT, TV, TVW, PBS+L, TT+L, TV+L, and TVW+L. d) Fluorescence images of CRT, HGMB, and PD‐L1 levels in Hepa1‐6 cells incubated with PBS, TT, TV, TVW, PBS+L, TT+L, TV+L, and TVW+L. Ex: 495 nm, Em: 519 nm. Scale bar: 100 µm. e–g) Quantification analysis of CRT, HGMB, and PD‐L1 in Hepa1‐6 cells incubated with PBS, TT, TV, TVW, PBS+L, TT+L, TV+L, and TVW+L. h) Relative viability of Hepa1‐6 cells after treatment with TT, TT+L, TV, TV+L, TVW, and TVW+L. i) The detection of apoptosis after different treatment groups (PBS, PBS+L, TT+L, TV+L, and TVW+L) by flow cytometry. Data were expressed as means ± SD. *p < 0.05, **p < 0.01, ***p < 0.001.

To date, anti‐PD‐1/PD‐L1 therapy has been widely used to break immunosuppression and reactivate the immune system. STAT3 plays a pivotal role in regulating PD‐L1, a crucial inhibitor that facilitates immune evasion. The inhibition of STAT3 could lead to a notable reduction in PD‐L1 expression and a substantial inhibition of tumor immune escape, consequently establishing an efficacious antitumor immune response. The STAT3 inhibitor WP1066 was used to regulate STAT3 expression in this research. The expression of STAT3, phosphorylated STAT3 and PD‐L1 after different treatments was studied by WB (**Figures** **3**a and S17, Supporting Information). After treating Hepa1‐6 cells with TT+L, TV+L, and TVW+L, there was a notable inhibition of phosphorylated STAT3 in the TVW+L group, which was attributed to the presence of WP1066 (Figure 3b). Meanwhile, the expression of PD‐L1 was reduced in all experimental groups following light exposure. It might be ascribed to the fact that following PDT, apoptotic tumor cells release tumor antigens that can be captured by DCs, while also releasing DAMPs to promote the maturation of immature DCs, resulting in an effective tumor‐specific immune response. Since TV could produce more ROS than TT after laser irradiation, TV was more effective to inhibit PD‐ L1 expression in the TV+L group than TT+L group. The lowest PD‐ L1 expression was observed in the TVW+L group, probably owing to the inhibition of STAT3 by WP1066. Specifically, the expression of PD‐L1 was reduced by 74% in the TVW+L group (Figure 3c).

To investigate whether PDT induced more pronounced ICD effects in combination with WP1066, the CRT and HMGB‐1 protein levels were measured by immunofluorescence (Figure 3d). Hepa1‐6 cells were incubated in equal concentrations of TT, TV, or TVW for 24 h, respectively, and two conditions of light and no light were set. Subsequently, the expression of CRT and HMGB‐1 in tumor cells was labelled by immunofluorescence. Calreticulin, a chaperone protein, acts as an “eat‐me” signal to stimulate the maturation of naive DCs and then induces an enhanced immune response when ER stress is generated. Therefore, increased expression of calreticulin is observed in response to enhanced immunogenicity, and exposure to CRT is a hallmark event in the development of ICD. CRT protein expression was significantly increased in the “With Light” group compared to the “Without Light” group. Furthermore, tumor cells incubated with TVW exhibited green fluorescence, with the highest signal value observed in the presence of the immunological drug WP1066. A quantitative analysis of the fluorescence intensity of each group revealed that the average fluorescence intensity of the TVW group was 1.44‐fold higher than that of the TV group under light conditions (Figure 3e). Under normal conditions, HMGB‐1 is mainly found within cells. However, in the event of an enhanced immunogenic response of tumor cells, there is a potential for increased release of HMGB‐1 from within the cell to the extracellular space. Extracellular HMGB‐1 can act as an immunostimulatory molecule, activating immune cells (e.g., macrophages, DCs, etc.) and thus initiating the immune response. Therefore, when the immunogenic response is enhanced, a reduction in intracellular HMGB‐1 expression is observed. As shown, the fluorescence signal value exhibited a decline in the “With Light” group in comparison to the “Without Light” group. And only a weak green fluorescence could be observed in the TVW+L group. After counting the fluorescence intensity of each group separately, it was observed that the expression of HMGB‐1 in the TVW group was reduced to 32% compared with the average fluorescence intensity of the TV group under the light condition (Figure 3f).

The immunotherapeutic efficacy of various treatments was further validated by detecting PD‐L1 levels using immunofluorescence. Hepa1‐6 cells were incubated in equal concentrations of TT, TV, or TVW for 24 h, and two conditions of “With Light” and “Without Light” were set, respectively. As shown, the levels of PD‐L1 in the “With Light” group were all decreased in comparison to the “Without Light” group. In addition, only weak green fluorescence of PD‐L1 was observed in the TVW+L group. Furthermore, after statistical analysis, the average fluorescence intensity in the TVW group decreased to 35.3% of that of the TV group (Figure 3g). The corresponding results demonstrated that PDT‐induced ICD enhanced the downregulation of PD‐L1 when combined with WP1066.

After confirming the multiple effects of VK3 in reducing oxygen consumption and WP1066 in amplifying immunogenic response, we further investigated whether TVW could improve the therapeutic effect of phototherapy. To demonstrate the therapeutic potential of TVW in phototherapy, we used the CCK‐8 assay to detect cell viability after different treatments. As shown in Figure 3h, TT, TV, and TVW exhibited a concentration‐dependent killing effect on Hepa1‐6 cells. As the concentration increased, TT, TV, and TVW showed enhanced killing effects on tumor cells. Cell survival was specifically analyzed when the concentration of THPP in the three nanoparticles was 1 µg mL^−1^. TT did not exhibit obvious inhibitory effect on the proliferation of Hepa1‐6 cells in the absence of light irradiation. Hepa1‐6 cells cultured with TT+L alone showed only a weak inhibitory effect on cell proliferation, and the survival rate was as high as 76.32%, which implied that TT‐triggered PDT was not sufficient to inhibit the proliferation of cancer cells. In the TV+L group, the cell survival rate was 61.47%, indicating that the introduction of VK3, a pyruvate kinase inhibitor, could effectively alleviate hypoxia and provide more “raw materials” for photodynamic therapy, thus enabling the generation of more ROS for PDT. Treatment of Hepa1‐6 cells with TVW under 660 nm laser irradiation revealed that the combined treatment group exhibited a reduction in the survival rate of cancer cells to approximately 22.76%, thereby achieving a potent antitumor therapy. The remarkable proliferation inhibition ability of TVW under laser irradiation may be attributed to the excellent glycolysis inhibition by VK3 and the potent down‐regulation of PD‐L1 by WP1066. In addition, the biocompatibility of TVW was studied using L929 as a normal cell line. As shown in Figure S18 (Supporting Information), TT, TV, and TVW did not exhibit any cytotoxicity to normal cells, implying excellent biocompatibility. Furthermore, the level of apoptosis in Hepa1‐6 cells after different drug treatments was quantified by flow cytometry using annexin V‐FITC/PI staining. As shown in Figure 3i, the untreated groups were unable to trigger apoptosis, regardless of whether or not they received laser irradiation. These findings indicate that light alone is an ineffective method for promoting tumor cell apoptosis. Incubation of Hepa1‐6 cells with TT+L resulted in a relatively low apoptosis level (33.9%), suggesting that PDT alone may not be sufficient to induce cell apoptosis. Due to the glycolysis inhibition effect of VK3, the apoptotic proportion of Hepa1‐6 cells was 51.7% in the TV+L group. In contrast, the apoptotic proportion of Hepa1‐6 cells was about 83.7% after treatment with TVW under 660 nm laser irradiation, which further indicated that PDT efficacy enhanced by VK3 and immunotherapy with WP1066 could effectively enhance apoptosis, which is expected to achieve a powerful combined photodynamic and immune antitumor therapy.

### 2.4 Evaluation of Therapeutic Effects of TVW In Vivo

**Figure 4:**
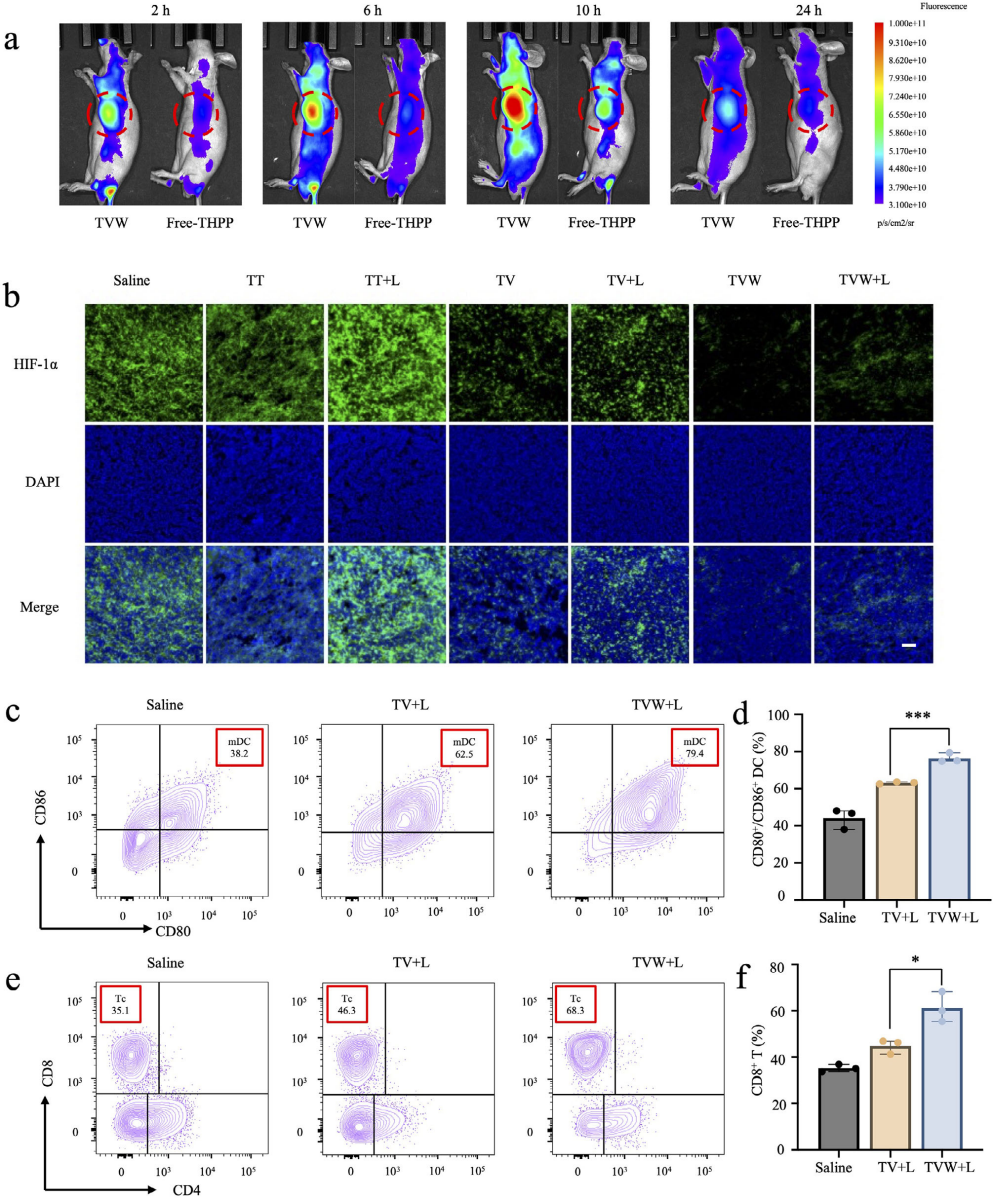
Therapeutic effect of TVW in vivo. a) In vivo biodistribution of free‐TVW and TVW in the Hepa1‐6 subcutaneous tumor model. b) CLSM images of Hepa1‐6 cells under HIF‐1α kit after incubation with saline, TT, TT+L, TV, TV+L, TVW, and TVW. Ex: 495 nm, Em: 519 nm. Scale bar: 50 µm. c) The detection of mature DCs (CD11c^+^/ CD80^+^/CD86^+^) after different treatment groups (PBS+L, TV+L, and TVW+L) by flow cytometry. d) The frequency of mature DCs in all collected DCs after the treatment. e) The detection of CD8+ T cell (CD3^+^/CD8^+^/CD4^−^) after different treatment groups (PBS+L, TV+L, and TVW+L) by flow cytometry. f) The frequency of CD8^+^ T lymphocytes in tumor. Data were expressed as means ± SD. *p < 0.05, **p < 0.01, ***p < 0.001.

Based on the fact that VK3 and WP1066 could enhance phototherapy and amplify immunotherapeutic efficacy in vitro, we further evaluated the anticancer performance of TVW in vivo. Due to the excellent fluorescence imaging ability of the photosensitizer THPP, the in vivo biodistribution of TVW was monitored in Hepa1‐6 tumor‐bearing C57 mice after injection of TVW and free‐THPP, respectively. The in vivo biodistribution was recorded by whole‐body fluorescence imaging at 2, 6, 10, and 24 h after the intravenous injection of TVW or free‐THPP into mice bearing Hepa1‐6 tumors. As illustrated in **Figure** **4**a, the intratumoral fluorescence signal of THPP was stronger in TVW treated group than free‐THPP. The results also indicated that TVW tended to accumulate in the tumor site and the fluorescence signal in the tumor site increased with time until reaching a peak after ≈10 h. We also analyzed the drug metabolism in major organs at different time points (2, 6, 10, and 24 h). The imaging results showed that TVW entered the systemic circulation via tail vein injection, with the drug primarily accumulating in the tumor tissue, while drugs in other organs were rapidly metabolized and eliminated (Figure S19, Supporting Information). Consistent with in *vivo* imaging results, the fluorescence intensity of TVW in the tumor reached its peak at 10 h. In addition, we also investigated the pharmacokinetics of TVW in Hepa1‐6 tumor‐bearing ICR mice. As shown in Figure S20 (Supporting Information), after treatment with TVW, the concentration of the photosensitizer in plasma decreased to ≈50% of the injected dose within 30 min.

Given that VK3 is capable of effectively reducing oxygen consumption by inhibiting glycolysis, an investigation was conducted to ascertain whether VK3 could alleviate tumor hypoxia in vivo. Hypoxia‐inducible factor 1α (HIF‐1α) antibody (ab308433) was used to assess tumor hypoxia levels. HIF‐1α is one of the commonly used markers of tissue hypoxia. Bright green fluorescence is observed when the tumor tissue is at hypoxic levels. On the contrary, the fluorescence intensity decreases as the hypoxia is alleviated. As illustrated in Figure 4b, the fluorescence signals of HIF‐1α in tumor treated with VK3‐containing nanoparticles (TV and TVW) exhibited a notable reduction, indicating that the hypoxia level of the tumor tissue was effectively alleviated.

In order to evaluate the potential synergistic effects of TVW‐based photodynamic immunotherapy, the immune response was then monitored by flow cytometry in order to gain insight into the activity of key immune cells. As PDT‐induced tumor‐associated antigens (TAAs) can be phagocytosed by immature DCs, this process further promotes DC maturation. Furthermore, mature DCs subsequently stimulate T lymphocytes to cause tumor cell killing by secreting toxic cytokines, including perforin, granzyme and interferon‐γ (IFN‐γ). The mice were randomly assigned to one of three groups. Once the tumors reached a volume of 200 mm^3^, the mice were injected with TV and TVW via the tail vein and subsequently irradiated with a 660 nm laser 10 h after the administration of the drugs. Tumor tissues were removed after 6 days of treatment. Flow cytometry was used to determine the frequency of mature DC (CD11c^+^/ CD80^+^/CD86^+^). As shown in Figure 4c, in comparison to the PBS+L group, TV+L and TVW+L experimental groups demonstrated an elevation in the prevalence of mature DCs, reaching 62.5% and 79.4%, respectively. The highest DC maturation frequency was exhibited by TVW+L, which indicates that photodynamic immunotherapy combination is effective in promoting DC maturation (Figure 4d). T‐cell typing was subsequently conducted using flow cytometry. As illustrated in Figure 4e,f, the frequency of CTL cells (CD3^+^/CD8^+^/CD4^−^) was increased to 46.3% and 68.3% in the TV+L and TVW+L experimental groups, respectively. The CTL frequency in the tumor tissues of the TVW+L group was the highest, which was 1.94‐fold higher than that of the PBS group.

**Figure 5:**
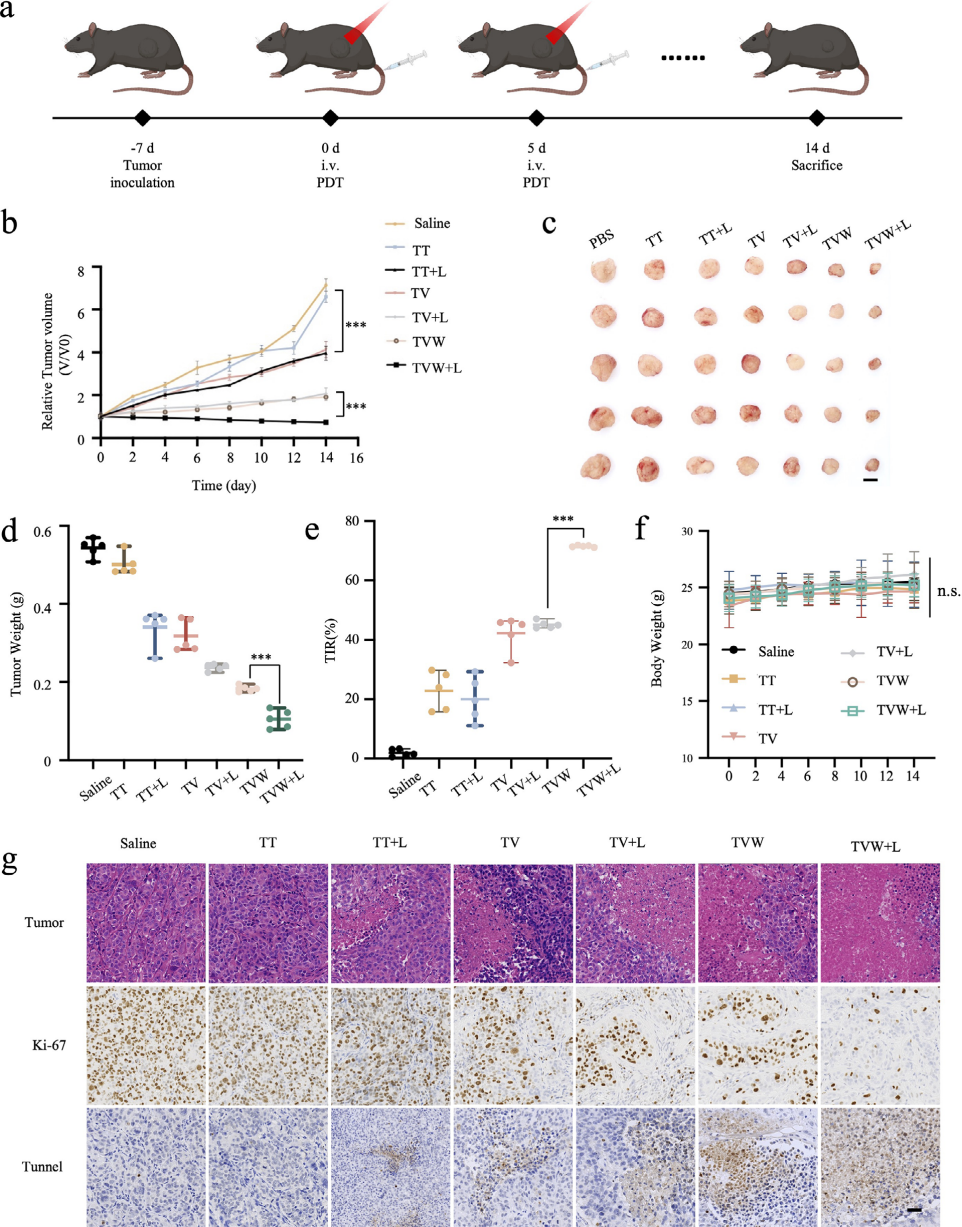
Synergistic antitumor of TVW in vivo. a) Schematic diagram of animal experiments. b) Tumor growth curves of Hepa1‐6 tumor‐bearing mice in different groups during the treatment period. c) Photographs of isolated tumors after 14 days of different treatments (*n* = 5). Scale bar: 1 cm. d) Changes in tumor weight in different treatment groups after 14 d (*n* = 5). e) Tumor inhibition rate of each treatment group (*n* = 5). f) The bodyweights of mice after different treatments (*n* = 5). g) The immunohistochemical analysis of tumor sections after different treatments by H&E, Ki67, and TUNEL. Scale bar: 100 µm. Data were expressed as means ± SD. *p < 0.05, **p < 0.01, ***p < 0.001.

At last, intravenous injection of various nanoparticles with a THPP equivalent dose of 3 mg kg^−1^ was performed in Hepa1‐6 tumor‐bearing mice to investigate their antitumor efficacy in vivo. Hepa1‐6 tumor‐bearing mice were randomly divided into seven groups (five mice per group) and injected with different formulations (saline, TT, TV, TVW, TT+L, TV+L, and TVW+L). Once the tumor volume reached ≈100 mm^3^, TT, TV, and TVW were injected into the mice through the tail vein on days 1 and 5. The tumor tissues in the TT+L, TV+L, and TVW+L treatment groups were irradiated with a 660 nm laser 10 h after the administration of the respective nanoparticles (**Figure** **5**a). The in vivo antitumor effect was evaluated by monitoring the changes in tumor volumes of Hepa1‐6 tumors following the injection of different nanoparticles (Figure 5b). A rapid increase in tumor volume was observed in the saline‐treated control group. TT alone had no significant inhibitory effect on tumor growth, while TT by 660 nm laser irradiation could inhibit tumor growth to a certain extent. The TV+L group demonstrated a notable inhibitory effect on tumor growth, which may be attributed to the introduction of VK3 in the covalent organic polymers. VK3 in TV effectively alleviated tumor hypoxia by reducing oxygen consumption, thereby providing more oxygen for PDT. The most favorable therapeutic outcomes were observed in the TVW combination therapy group following laser irradiation. It might be attributed to the inhibitory effect of WP1066 on pSTAT3, which amplified the immunogenic response induced by PDT. The combination of enhanced photodynamic therapy and an amplified immune response resulted in the maximum killing effect on tumor cells. Mice were sacrificed after 14 days of treatment. Tumor tissue was collected and weighed (Figure 5c). The weights of the tumors following treatment with saline, TT, TV, TVW, TT+L, TV+L, and TVW+L were 0.54, 0.50, 0.34, 0.32, 0.24, 0.18, and 0.10 g, respectively (Figure 5d). The optimal therapeutic effect of TVW+L was also demonstrated by calculating the tumor inhibition rate, which reached 71.5%. This was significantly higher than that of the other treatment groups (Figure 5e). The body weights of the mice were also monitored during the course of treatment. As shown in Figure 5f, the weights of the mice in all experimental groups remained relatively stable.

To ascertain the inhibitory effect of different drug treatments on tumor tissues, tumor tissue sections were analyzed using hematoxylin and eosin (H&E) staining, Ki‐67, and TUNEL immunohistochemical analysis. As shown in Figure 5g, more apoptosis was induced in the group treated with the 660 nm laser compared to the “without light” group. In the illuminated group, the highest level of apoptosis in tumor tissues was observed in the combined TVW+L treatment group (Figure S21, Supporting Information). Meanwhile, TUNEL immunohistochemical analyses showed the same results, with lower tumor cell proliferation generally observed in the illuminated treatment groups and the lowest level of proliferation in the combined TVW+L group (Figure S22, Supporting Information). These results further suggested that TVW exhibited excellent antitumor activity under laser irradiation.

### 2.5 Biosafety Evolution In Vivo

**Figure 6:**
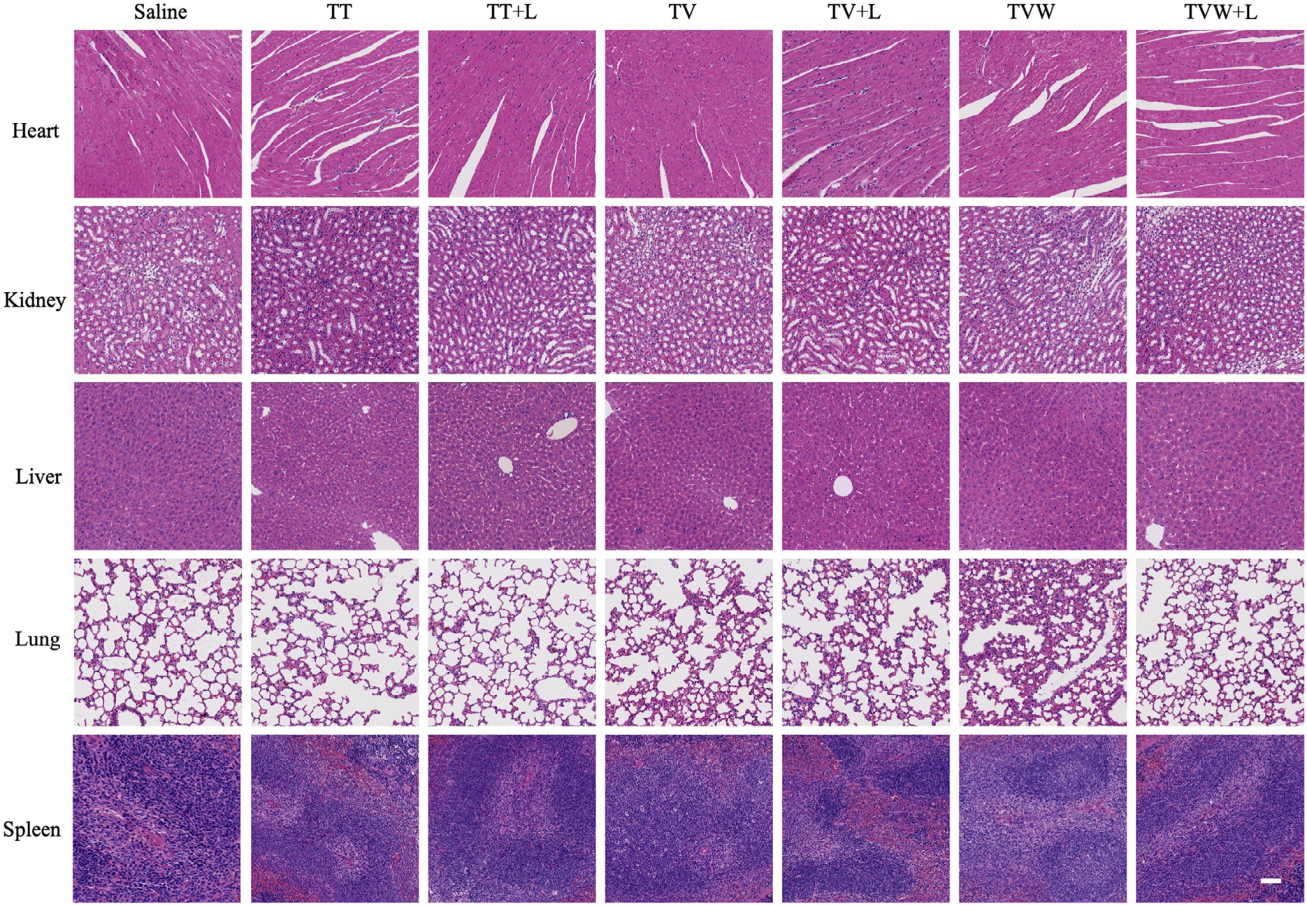
Biosafety of nanomedicine. H&E staining of heart, liver, spleen, lung, and kidney after 14 days of the corresponding treatment. Scale bar: 100 µm.

Subsequently, the biosafety of COPs was evaluated. First, the results of the hemolysis test revealed that the hemolysis rate of different treatment groups was lower than 2% (Figure S23, Supporting Information), which verified the biosafety of the drug‐loaded COPs in the circulation. By H&E staining of the heart, liver, spleen, lungs, and kidneys of mice from different treatment groups, none of them showed significant tissue damage or toxicity (**Figure** **6**). Finally, routine blood indices, including red blood cells, white blood cells, and platelets, were evaluated. The findings revealed no statistically significant differences between the various treatments (Figures S24 and S25, Supporting Information). The aforementioned results suggested that COPs exhibited excellent biosafety.

## 3 Conclusion 

In summary, we successfully synthesized an acid‐responsive nanosized COP composed of photosensitizer porphyrin and PK inhibitor VK3, which could be used to encapsulate the STAT3 inhibitor WP1066. After TVW accumulated in tumor tissue, it would be dissociated to release VK3 and WP1066 owing to the acidic tumor microenvironment. The photosensitizer THPP exhibited photodynamic cytotoxicity when exposed to light at 660 nm. The PK inhibitor VK3 was capable of reducing oxygen consumption by inhibiting glycolysis, leading to the relief of the hypoxic tumor microenvironment. Therefore, more ROS could be produced in TVW‐based PDT, thereby enhancing the efficacy of PDT treatment. Meanwhile, the hydrolysis of pH‐sensitive COPs enabled the efficient release of WP1066. WP1066 downregulated the level of PD‐L1 through the inhibition of STAT3, which further inhibited the binding of PD‐L1 and PD‐1, thereby providing effective tumor immunotherapy. The results of both in vitro and in vivo experiments demonstrated the promising tumor‐suppressive effect of TVW‐based photodynamic immunotherapy. This study presents a potential application of acid‐responsive nanoscale COPs, which may simultaneously enhance PDT and amplify immunotherapeutic efficacy.

## 4 Experimental Section

### Materials

Acryloyl chloride, meso‐tetra (*p*‐hydroxyphenyl)‐porphyrin (THPP), and anhydrous dimethyl sulfoxide (DMSO) were obtained from J&K Chemical Co. 4,4′‐trimethylene dipiperidine (TMPD) was purchased from TCI. Vitamin K3 and WP1066 were purchased from Shanghai Aladdin Biochemical Technology Co., Ltd. Mono‐amino‐terminated poly (ethylene glycol) (mPEG5k‐NH_2_) was obtained from Chongqing Yuying Pharmaceutical Technology Co., Ltd. Dulbecco's modified Eagle medium (DMEM) was obtained from Gibco Life Technologies (USA). Fetal bovine serum (FBS) was offered by Zhejiang Baidi Biotechnology Co., Ltd. Reactive oxygen species assay kit (DCFH‐DA) and ATP assay kit were provided by Beyotime Biotechnology (Shanghai, China). CCK8 assay kit was provided by Meilun Biotechnology Co., Ltd. (Jiangsu, China). Oxygen Consumption Assay Kit was obtained from Shanghai Kanglang Biotechnology Co., Ltd. (Shanghai, China). Pyruvate activity assay kit was purchased from Abcam Plc. (USA). Anti‐calreticulin antibody (bs‐5913R) was supplied by Bioss (Beijing, China). Anti‐HMGB‐1 antibody (ab79823) and anti‐PD‐L1 antibody (ab213480) were bought from Abcam (Cambridge, UK).

### Characterization

The ultraviolet–visible spectra were obtained using a UV–vis spectrometer (UV‐2550, Shimadzu). The hydrodynamic diameter and size distribution of COPs were characterized at 25 °C with a 633 nm He‐Ne laser using DLS measurements (Zetasizer Nano‐ZS, Malvern Instruments), and the zeta potential was also measured with Zetasizer Nano‐ZS. The morphology of the nanoparticles was observed by TEM. Fluorescence images were obtained via an inverted fluorescence microscope (Nikon Ti2, Japan).

### Synthesis of Porphyrin‐Containing COPs (Denoted as TT)

First, THPP (0.1 mmol) and triethylamine (1.2 mmol TEA) were added to 50 mL of anhydrous tetrahydrofuran (THF). Following this, acryloyl chloride (1.2 mmol) was slowly dripped into the solution under nitrogen atmosphere at 0 °C. Then the THF was removed through rotary evaporation to obtain crude product. The crude product was re‐dissolved in dichloromethane and subsequently washed with aqueous Na₂CO_4_ solution for three times. Finally, the product was washed with distilled water for three times. After the organic phase had been dried with anhydrous Na₂CO_4_ for 2 h, it was concentrated by rotary evaporation. The concentrate was precipitated in cold hexane, and the solid was obtained by centrifugation. Subsequently, it was dried in a vacuum oven at 45 °C for 24 h to yield acryloyl‐THPP. The Michael addition reaction between acryloyl‐THPP (0.5 mmol) and 4,4′‐trimethylenebipiperidine (0.5 mmol, TMDP) was conducted in anhydrous DMSO at 60 °C for 24 h. The reaction was continued by adding mPEG5k‐NH_2_ (0.5 mmol) for 36 h. The product was dialyzed with a mixture of Milli‐Q water and ethanol (ethanol concentrations of 100%, 75%, 50%, 25%, and 0) for 48 h. The final product TT was obtained after lyophilization.

### Synthesis of Porphyrin and VK3‐Containing COPs (Denoted as TV)

First, VK3 (0.2 mmol) was dissolved in THF solution. Subsequently, under a nitrogen atmosphere and in an ice‐water bath at 0 °C, hydrazine hydrate (0.6 mmol) was added dropwise to the solution. After maintaining 0 °C and stirring for 2 h, the excess solvent was removed by rotary evaporation, and the product was obtained by freeze‐drying to yield NH_2_‐VK3. Acryloyl‐THPP (0.05 mmol) was stirred with NH_2_‐VK3 (0.1 mmol) at 60 °C for 24 h in DMSO. The reaction was then continued with the addition of mPEG5k‐NH_2_ for a further 36 h. The product was dialyzed with a mixture of Milli‐Q water and ethanol (ethanol concentrations of 100%, 75%, 50%, 25%, and 0) for 48 h, and finally lyophilized to give the product TV.

### Synthesis of WP1066 Encapsulated TV (denoted as TVW)

After the synthesis of TV, WP1066 (0.005 mmol) was added to continue stirring for 24 h. The product TVW was obtained after lyophilization. Removal of excess WP1066 by ultracentrifugation (10 000 r min^−1^).

### In Vitro Drug Release

The TVW solution (1 mL) was placed in a dialysis bag with a molecular weight cut‐off (MWCO) of 8000–14000 and then submerged in 10 mL of phosphate‐buffered saline (PBS) (pH 7.4) and PBS (pH 6.0) containing 1% (w v^−1^) Tween‐80 and placed on a shaker at 37 °C. The solution outside the dialysis bag was sampled at 0.5, 1, 2, 4, 6, 10, and 24 h, respectively. The concentration of drug in the solution was quantified by UV–vis absorption spectroscopy, utilizing drug‐specific absorption peaks.

### Cell Lines

Liver cancer Hepa1‐6 cells were obtained from Keygen Biotech Co., Ltd. (Jiangsu, China). Hepa1‐6 cells were cultured in DMEM (Wisent, Nanjing, China) containing fetal bovine serum (FBS), penicillin (100 units mL^−1^), and streptomycin (100 mg mL^−1^) in a 37 °C incubator with 5% CO_2_ (Thermo Fisher, Waltham, USA).

### Cellular Uptake In Vitro

To study the cellular uptake of drugs, the Hepa1‐6 cells were cultured in 24‐well plates for 24 h at a density of 2 × 10^4^ cells per well. Subsequently, a fresh medium containing TVW was added to the culture medium and treated for 1, 2, and 4 h. The hepa1‐6 cells were then washed three times with PBS. Following a 20 min incubation during which the cells were stained with DAPI, the intracellular fluorescence intensity was observed under an inverted fluorescence microscope.

### Detection of ROS Levels In Vitro

The ability of TT to generate ROS was evaluated using the DCFH‐DA probe. Hepa1‐6 cells were seeded into 24‐well plates at a density of 5 × 10^4^ cells per well. After incubation for 24 h, the medium was replaced with fresh medium containing TVW for 4 h, and the plates were then stained with the DCFH‐DA probe for 30 min before being washed three times with PBS. Fluorescence imaging of the cells was observed using the inverted fluorescence microscope following light exposure.

### Detection of Pyruvate Kinase Levels In Vitro

To detect pyruvate kinase, Hepa1‐6 cells were cultured in 24‐well plates at a density of 1 × 10^4^ per well for 24 h. Subsequently, Hepa1‐6 cells were treated with PBS, TT, TV, and TVW for 12 h. The cells were then harvested and washed three times in PBS. Following the manufacturer's specifications, the pyruvate kinase activity assay kit was utilized to quantify the pyruvate kinase enzyme's activity.

### Detection of Intracellular ATP Levels In Vitro

To evaluate the ability of VK3 to inhibit the synthesis of ATP, the ATP assay kit (Beyotime, China) was used. Hepa1‐6 cells were seeded into 24‐well plates at a density of 4 × 10^4^ per well and incubated for 24 h. The cells were then treated with PBS, TT, TV, and TVW for 12 h. The intracellular ATP level was evaluated via the ATP assay kit. Furthermore, the fluorescence signals were quantified by chemiluminescence, and the relative intracellular ATP concentration was calculated using the following Equation (1):

(1) $$\mathrm{Relative}\;\mathrm{ATP}\;\mathrm{level}\left(\%\right)=\left(F_{\mathrm{sample}}-F_{\mathrm{blank}}\right)/\left(F_{\mathrm{control}}-F_{\mathrm{blank}}\right)\times 100$$

where *F*_sample_ is the fluorescence signal of the group of samples, *F*_blank_ is the fluorescence signal of the blank group, and *F*_control_ is the negative control group.

### Detection of Oxygen Consumption In Vitro

To assess the OCR, Oxygen Consumption Assay Kit (Shanghai Kanglang Biotechnology Co., Ltd.) was used. Hepa1‐6 cells were seeded into 96‐well plates at 1 × 10^4^ per well and incubated for 24 h. The samples were then treated for 12 h in PBS and the medium containing TT, TV, and TVW. The oxygen consumption rate was detected according to the manufacturer's instructions.

### Western Blot Analysis

The pyruvate kinase level was quantified following different treatments using a Western blot assay. Hepa1‐6 cells were cultured in six‐well plates at a density of 1 × 10^6^ cells per well in DMEM. After incubation at 37 °C under 5% CO₂ for 24 h, the cells were treated with PBS, TT, TV, and TVW for a further 4 h. Following three washes with PBS, the cells were irradiated with a 660 nm laser (0.3 W cm^−2^, 5 min). Following three washes with PBS, the cells were harvested by trypsinization. The total protein content was quantified using a bicinchoninic acid (BCA) protein quantification kit. Following the transfer to the polyvinylidene fluoride membrane, the 10% sodium dodecyl sulfate polyacrylamide gel electrophoresis (SDS‐PAGE) was used to separate the proteins in each sample. The membrane was incubated with T‐TBS containing 5% bovine serum albumin (BSA) for 1 h and then incubated with relevant primary antibody anti‐pyruvate kinase at 4 °C overnight. Then, the membranes were washed with T‐TBS three times, and 12 hybridized with goat anti‐Rabbit IgG(H+L) as a secondary antibody against pyruvate kinase primary antibody at 25 °C for 1 h. The membranes were visualized on X‐ray films and detected by chemiluminescence using SuperSignal West Dura Extended Duration Substrate.

### Immunofluorescence Analysis

To assess the levels of related proteins (CRT and HMGB‐1) and to investigate the ICD response induced by TT, TV, and TVW, immunofluorescence was used. In addition, the inhibitory effect of these compounds on PD‐L1 was also evaluated. Hepa1‐6 cells were cultured at a density of 8 × 10^4^ per well in six‐well plates for 24 h. The cells were then incubated with PBS, TT, TV, and TVW for 6 h. The light groups were irradiated with a 660 nm laser (0.3 W cm^−2^, 5 min), and incubated in the dark at 37 °C for 2 h. The supernatant was then discarded, and the cells were incubated with 0.3% Triton X‐100 for 15 min after washing three times with PBS. The cells were then treated with 5% BSA for 30 min. After removal of the supernatant, cells were incubated with the primary antibody at 4 °C overnight. The cells were incubated with a fluorescent secondary antibody for 1 h, and DAPI staining was used to identify the nuclei of the cells. The fluorescence was observed by an inverted fluorescence microscope (Nikon Ti2, Japan).

### Cytotoxicity Assay In Vitro

Cell Counting Kit‐8 (CCK‐8) was used to evaluate the cytotoxicity of the nanomaterials at different concentrations. The Hepa1‐6 cells (density of 8 × 10^3^ per well) were seeded into 96‐well plates. 200 µL DMEM was added as the culture medium. The cells were incubated for 24 h. Thereafter, TT, TV, and TVW were added at different concentrations for 4 h. The light group was irradiated with a laser (660 nm, 0.3 W cm^−2^) for 5 min, after which the incubation was continued until 24 h. The medium was then changed to DMEM medium containing 10% CCK‐8, and the cells were incubated for a further 4 h at 37 °C.

### Subcutaneous Hepatoma Model and Biosafety Evaluation

All animal experiments were conducted following the guidelines of Institutional Animal Care and Use Committee, Zhejiang Center of Laboratory Animals (ZJCLA) and the “Principles of Laboratory Animal Care” (NIH publication no. 86‐23, revised 1985). The assigned approval number was ZJCLA‐IACUC‐20010619. The authors state that the animal experiments conformed with the Helsinki Declaration of 1975, as revised in 2008 (5) concerning Human and Animal Rights. The antitumor efficacy of the COPs was evaluated on subcutaneous hepa1‐6 xenograft tumor models. Healthy male C57 mice (20 ± 2 g, 4–5 weeks old) were purchased from Zhejiang Academy of Medical Sciences. Hepa1‐6 cells (1 × 10^6^) were injected subcutaneously into the right flank of mice. When the tumors reached about 100 mm^3^, the mice were randomly divided into seven groups (Saline, TT, TT+L, TV, TV+L, TVW, and TVW+L). TT, TV, TVW were injected into the tail vein. 4 h later, the groups with light were locally irradiated by 660 nm laser (0.3 W cm^−2^) for 5 min. The tumor volume and body weight of mice were calculated and recorded regularly to assess the antitumor efficacy of drugs. After 14 d, the mice were euthanized, and the tumors of each group were weighted and recorded by a digital camera. Blood tests and H&E were used to verify the biosafety of nanodrugs.

The tumor volume was calculated according to the following equation (2):

(2) $$\mathrm{Volume}=\left(\mathrm{Tumor}\;\mathrm{Length}\right)\times {\left(\mathrm{Tumor}\;\mathrm{Width}\right)}^{2}/2$$

### Body Imaging and Biodistribution In Vivo

Hepa1‐6 subcutaneous tumor‐bearing mice were divided into two groups randomly and injected with TVW and free‐THPP, respectively. At the predetermined time, in vivo body fluorescent images were taken by an in *vivo* imaging system by collecting the TVW signals. The metabolism within vital organ was also explored by monitoring fluorescence imaging of each organ in the body (heart, liver, spleen, lungs, and kidneys) at different time points.

### Pharmacokinetic Studies In Vivo

To obtain the pharmacokinetics profiles, Hepa1‐6 tumor‐bearing ICR mice were intravenously injected with TVW. At different time points (0.033, 0.5, 1, 3, 6, 12, and 24 h), blood (100 µL) was collected. The plasma was obtained after 2000 rpm for 15 min and diluted with 1 × PBS (100 µL). Then, the fluorescence intensity of TVW was measured.

### Flow Cytometry

After 14 days of different treatments, mice were sacrificed. The tumor was first stripped and placed in a complete medium containing DMEM+10% and subsequently maintained on ice. Approximately 0.1 g of tumor was transferred to a 1.5 mL EP tube, which had been previously filled with 200 µL of complete medium. The tumor was then minced with scissors and digested in 5 mL of DMEM containing 0.1 mg mL^−1^ DNase‐I and 1 mg mL^−1^ collagenase IV. After 1 h at 37 °C, the residual material that had not been fully digested was removed from the cell suspension using 70‐µm strainer. For intracellular cytokine staining, it is essential to first stimulate the cells with Cell Activation Cocktail (with Brefeldin A) before staining the cell suspension for both live and dead cells. Following the staining of cell membranes with antibodies, fixation, and permeabilization were performed, and cell suspensions were stained with anti‐IFN‐γ and anti‐Granzyme B monoclonal antibodies. After 24 h, the residual dye was washed away with PBS and the suspension was filtered again through a 40‐µm strainer to obtain a final single‐cell suspension. Single cell suspensions were detected using a BD LSR Fortessa flow cytometer (BD Biosciences, NJ, USA). Data were analyzed using FlowJo software (Tree Star, Ashland, OR).

### Immunofluorescence Staining

14 days after the different treatments, mice were sacrificed. The tumor was taken in 4% paraformaldehyde for fixation for 48 h. Following paraffin embedding, the sections were dewaxed with water, xylene, and various concentrations of ethanol (100%, 95%, 85%, and 75%). Finally, antigen recovery and blocking were conducted. Subsequently, the manufacturer's instructions for the relevant products were followed, with the sections undergoing a primary antibody (anti‐CD4, anti‐CD8, or anti‐IFN‐γ) treatment for 1 h at 37 °C and then the secondary antibody treatment for 30 min. The fluorescence was observed by an inverted fluorescence microscope (Nikon Ti2, Japan).

### Statistics Analysis

The data were presented as mean ± standard deviation (SD). The statistical differences were analyzed by Student's *t*‐test and one‐way analysis of variance (ANOVA) with Tukey's post‐hoc test by GraphPad Prism 9.5.0. This symbol of n.s. indicates no significant difference. *P < 0.05, **P < 0.01, ***P < 0.001.

## Conflict of Interest

The authors declare no conflict of interest.

## Ethics Approval and Consent to Participate

All animal experiments were conducted following the guidelines for Institutional Animal Care and Use Committee, Zhejiang Center of Laboratory Animals (ZJCLA) and the “Principles of Laboratory Animal Care” (NIH Publication No. 86‐23, revised 1985). The assigned approval number was ZJCLA‐IACUC‐20010619.

## Supporting information



Supporting Information

## Data Availability

The data that support the findings of this study are available from the corresponding author upon reasonable request.
