# Supplementary material for: A pH‐Sensitive Nanosized Covalent–Organic Polymer for Enhanced Tumor Photodynamic Immunotherapy by Hypoxia Relief and STAT3 Inhibition
Source: Adv Sci (Weinh). 2025 May 14;12(29):e04860. doi: 10.1002/advs.202504860 (PMC12362755; doi:10.1002/advs.202504860)
Supplement: Supplementary file 1 — Supporting Information [file ADVS-12-e04860-s001.docx]

((Supporting Information can be included here using this template))

Supporting Information

A pH-Sensitive Nanosized Covalent Organic Polymer for Enhanced Tumor Photodynamic Immunotherapy by Hypoxia Relief and STAT3 Inhibition

Lei Lei, Wenbin Dai, Jinchao Zhao, Angfeng Jiang, Haisheng Peng, Qiao Jin*, Xiaojing Li*, Zhe Tang*

Lei Lei, Jinchao Zhao, Angfeng Jiang

Department of General Surgery, The Fourth Affiliated Hospital, International Institutes of Medicine, Zhejiang University School of Medicine, Yiwu, 322000, China

Wenbin Dai, Qiao Jin

MOE Key Laboratory of Macromolecular Synthesis and Functionalization, Department of Polymer Science and Engineering, Zhejiang University, Hangzhou, 310058, China

E-mail: [jinqiao@zju.edu.cn](mailto:jinqiao@zju.edu.cn) (Q. Jin)

Haisheng Peng

Department of Pharmacology, Medical College of Shaoxing University, Shaoxing 312099, China

Xiaojing Li

Department of Gynecology, The Second Affiliated Hospital, School of Medicine, Zhejiang University, Hangzhou 310058, China

E-mail: xiaojingli@zju.edu.cn (X. Li)

Zhe Tang

Department of General Surgery, The Fourth Affiliated Hospital, International Institutes of Medicine, Zhejiang University School of Medicine, Yiwu, 322000, China

Department of Surgery, The Second Affiliated Hospital, School of Medicine, Zhejiang University, Hangzhou, 310058, China

E-mail: 8xi@zju.edu.cn (Z. Tang)


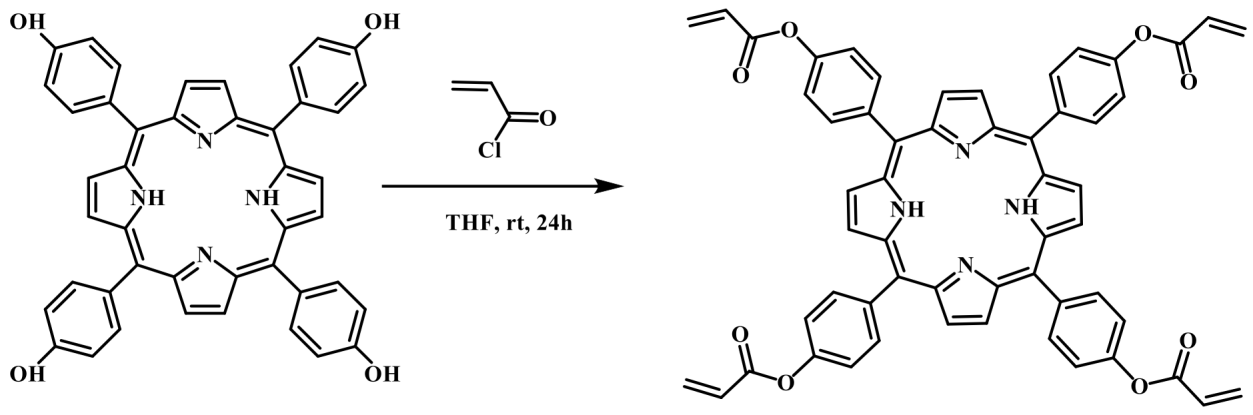


Figure S1: Schematic illustration of the preparation and structure of acryloyl -THPP.


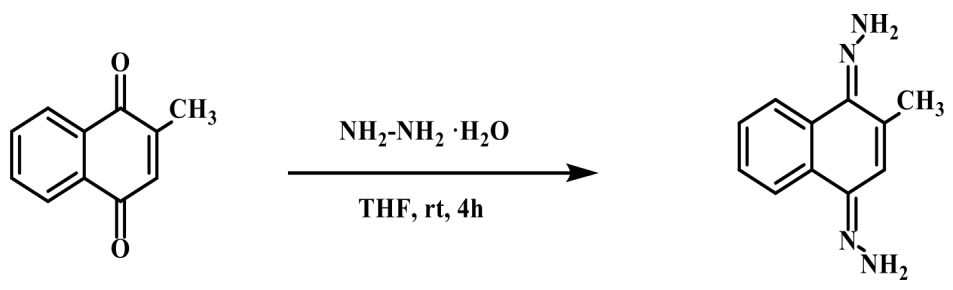


Figure S2: Schematic illustration of the preparation and structure of NH_2_-VK3.


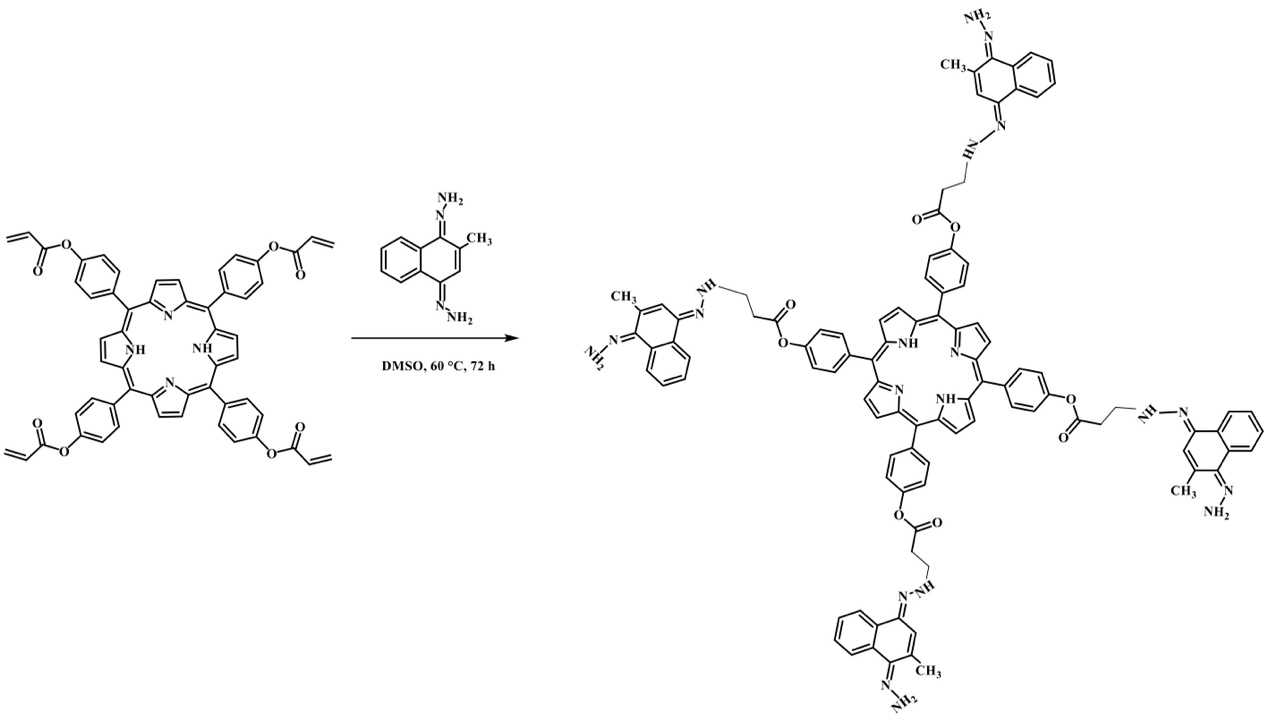

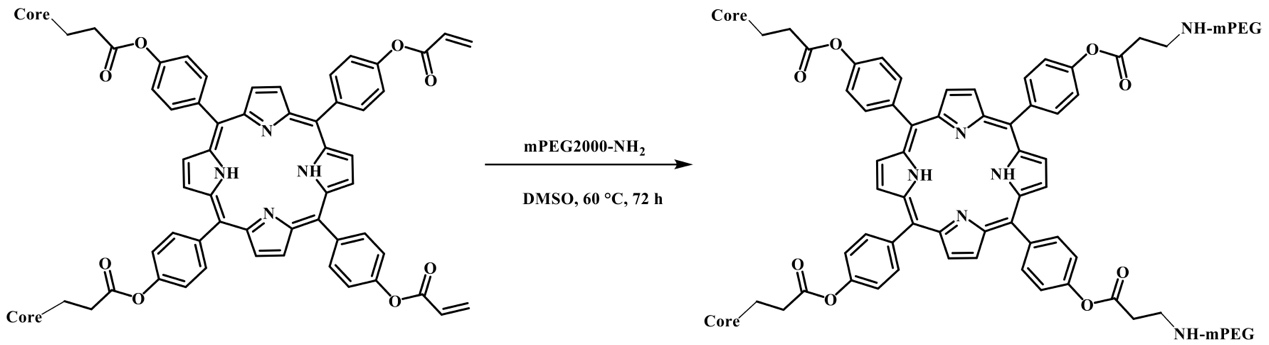


Figure S3: Schematic illustration of the preparation and structure of TVW.


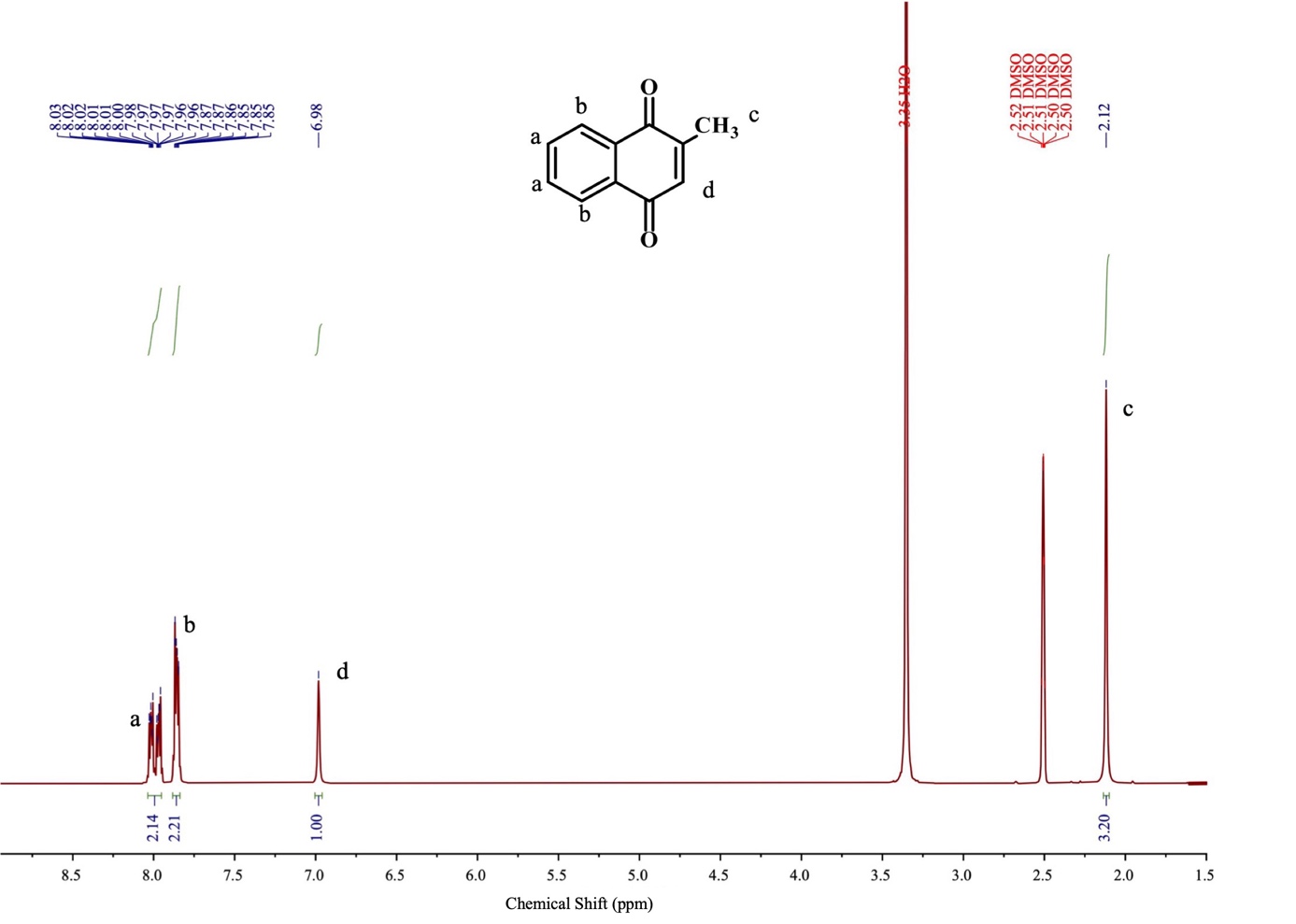


Figure S4: ^1^H NMR spectrum of VK3.


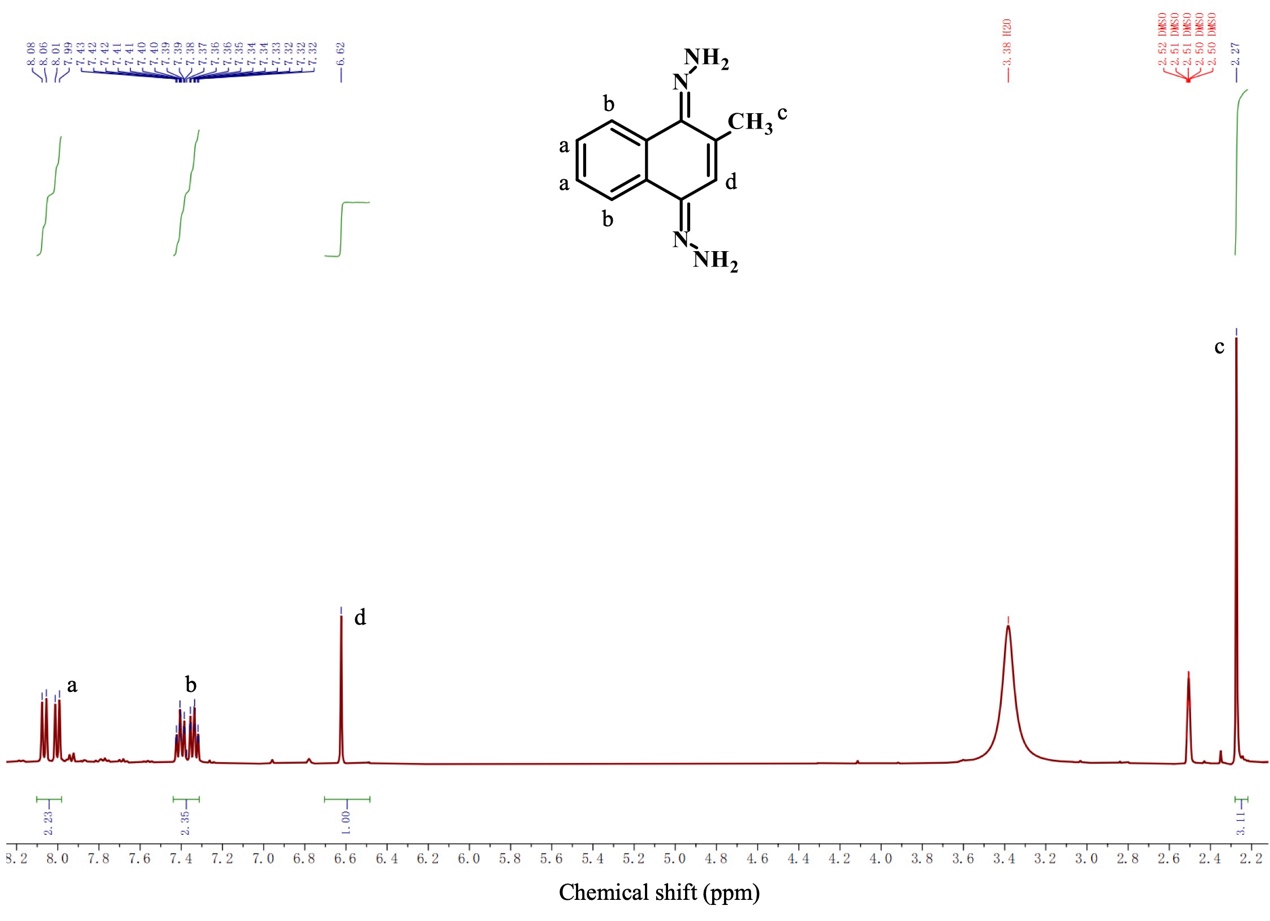


Figure S5: ^1^H NMR spectrum of NH_2_-VK3.


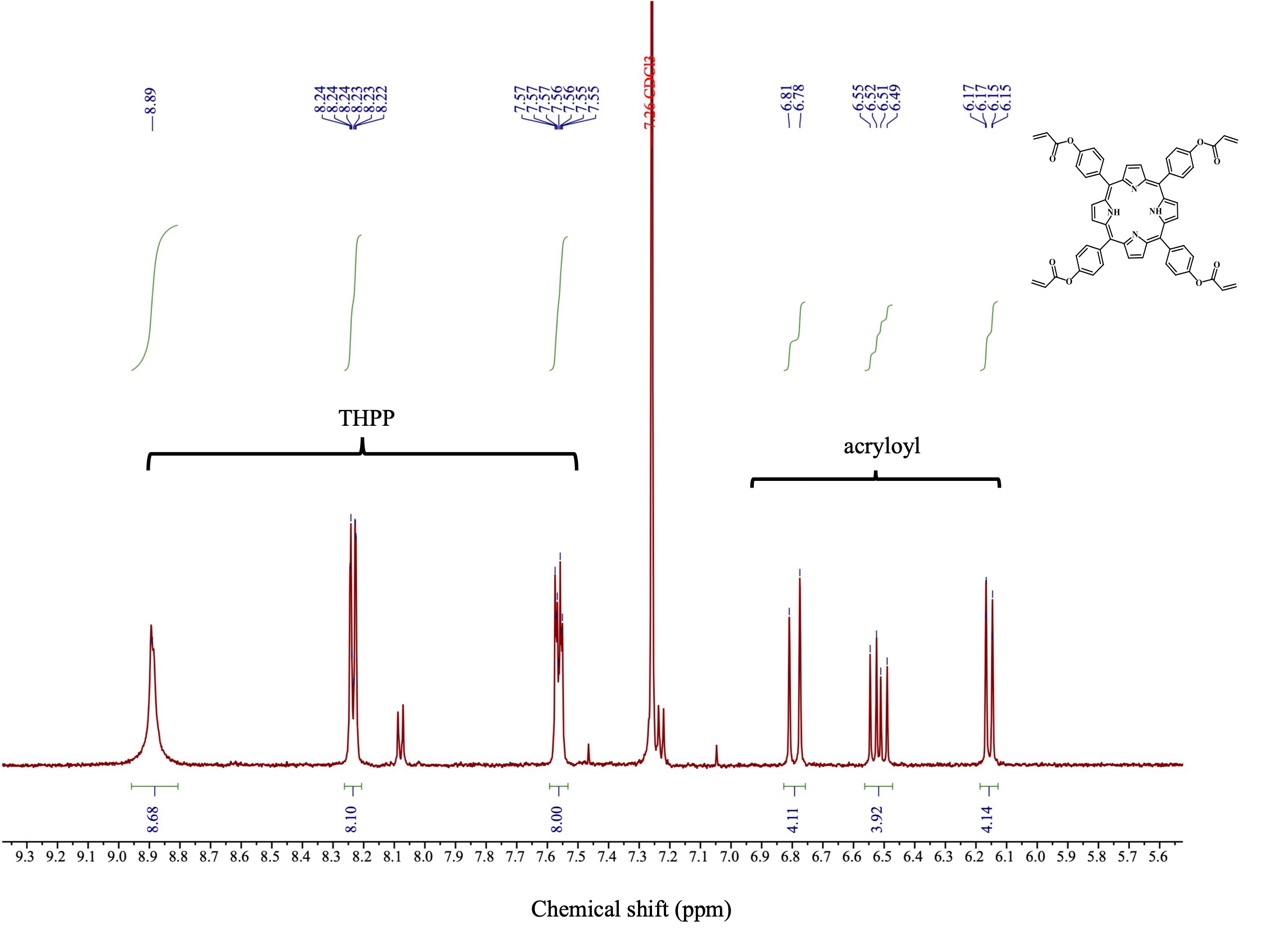


Figure S6: ^1^H NMR spectrum of acryloyl-THPP.


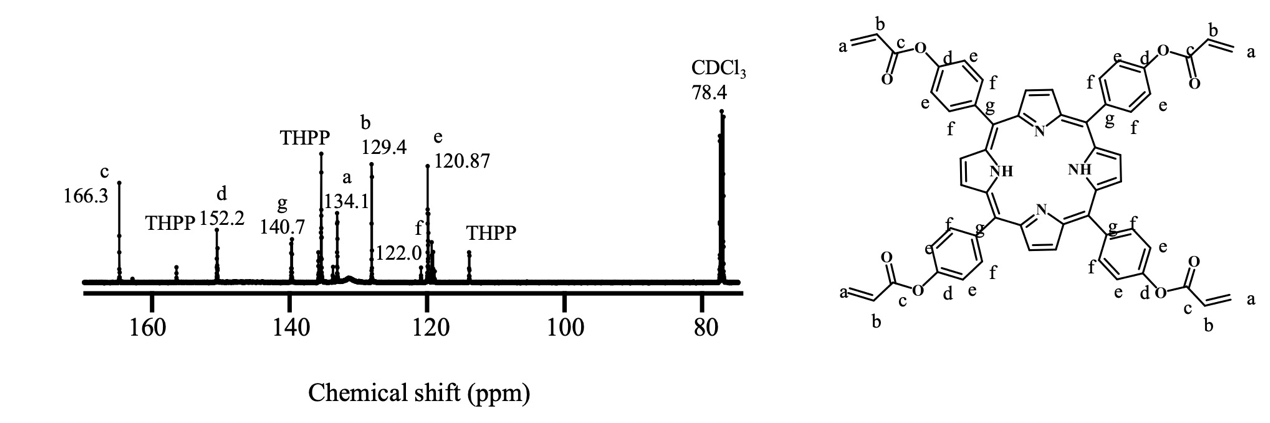


Figure S7: ^13^C NMR spectrum of acryloyl-THPP.


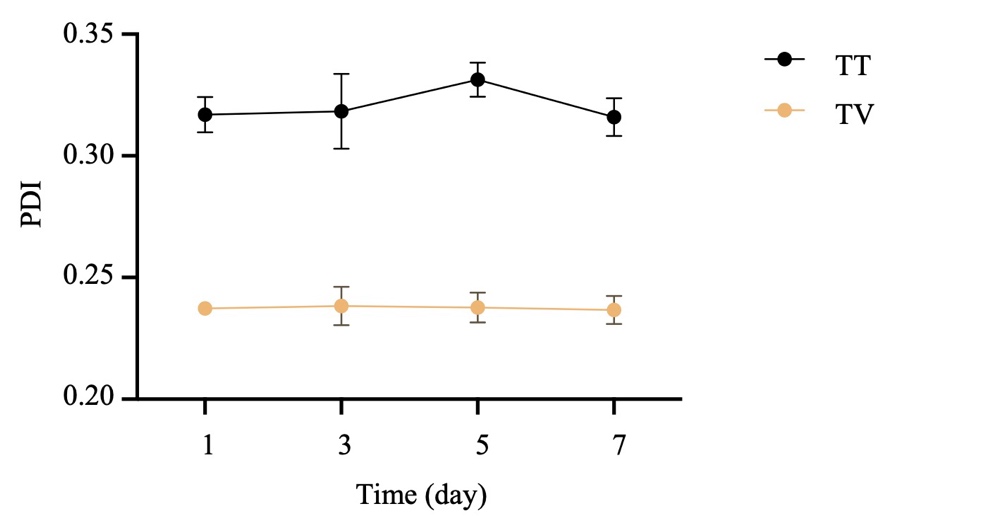


Figure S8: PDI changes of TT and TV upon incubating in PBS for 7 days.


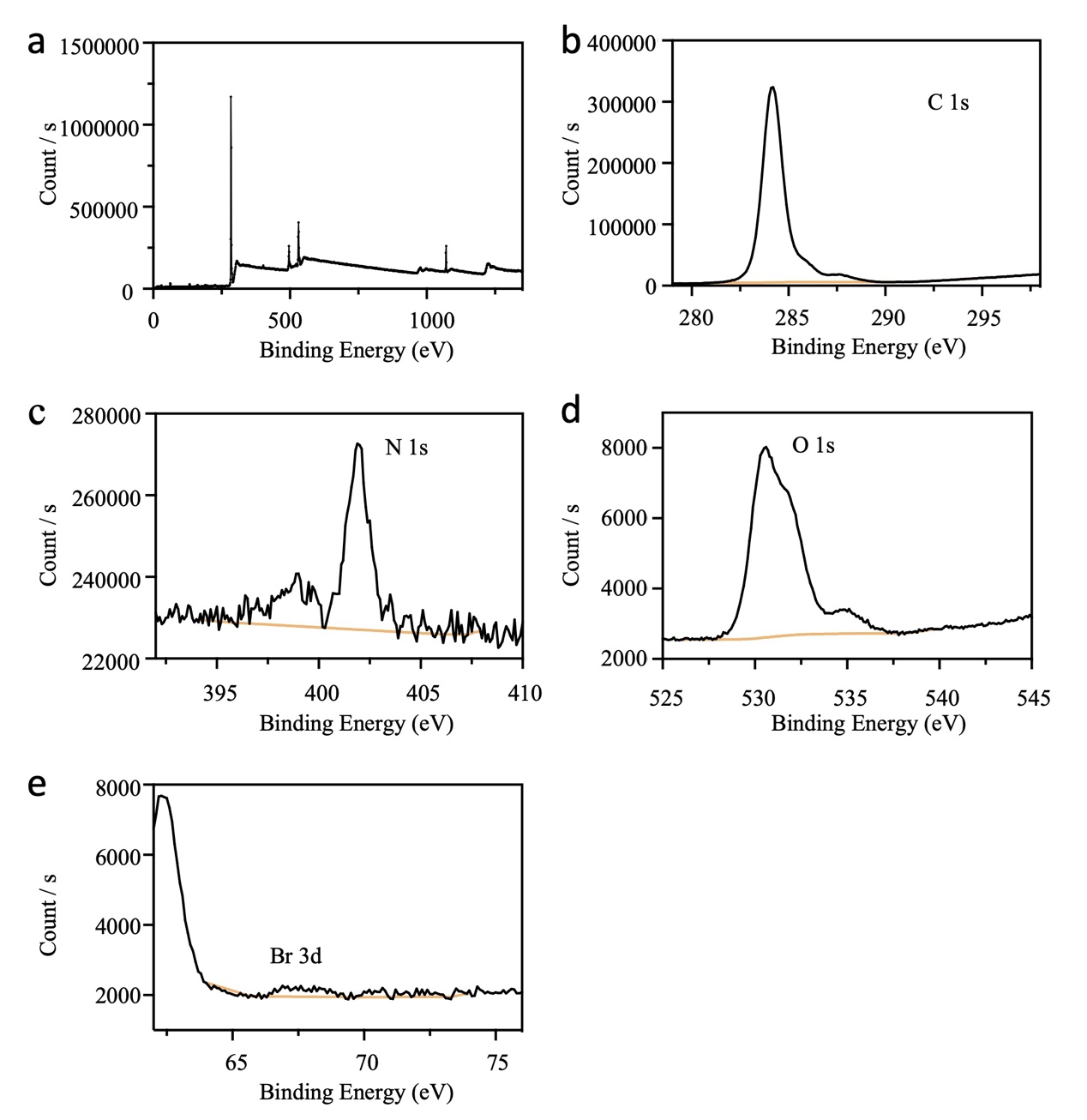


Figure S9: (a-e) The XPS spectra of TVW.


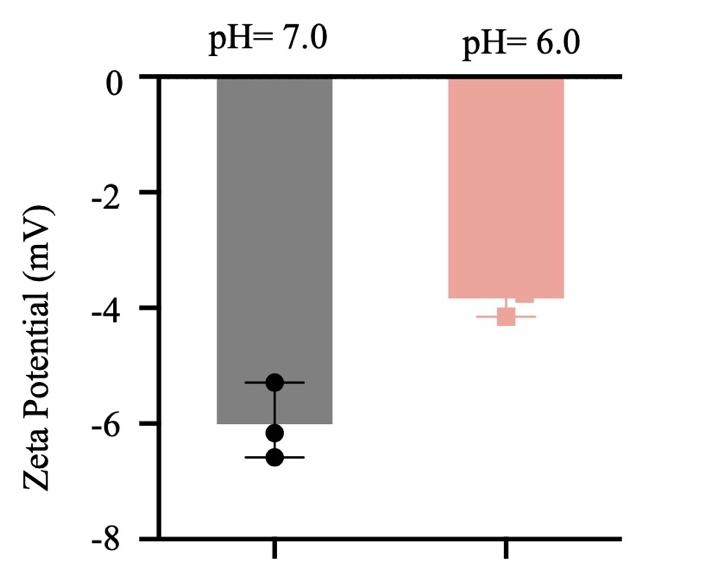


Figure S10: Zeta potential of TVW in pH 7.0 and pH 6.0 buffer.


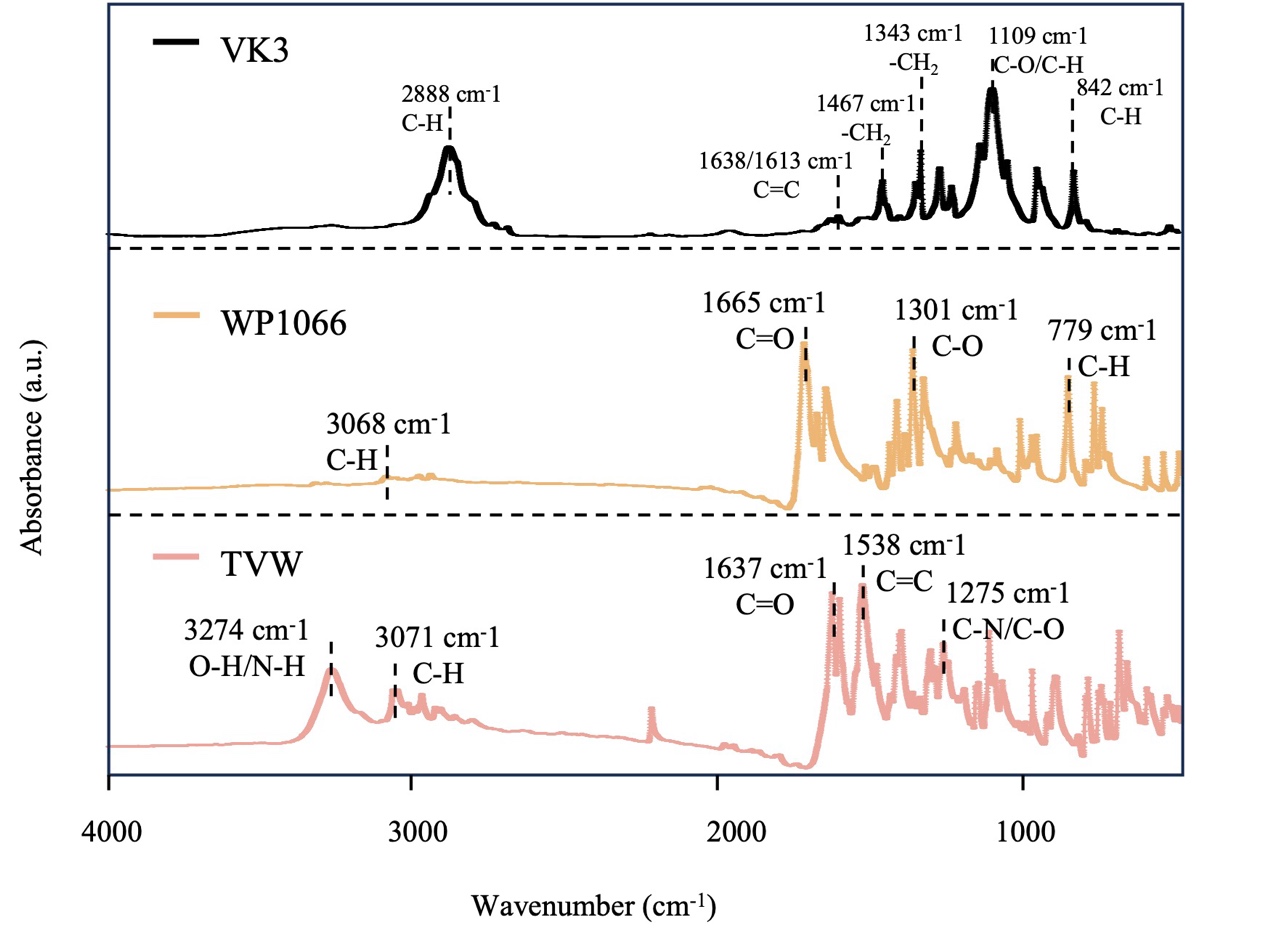


Figure S11: FT-IR of VK3, WP1066 and TVW samples.


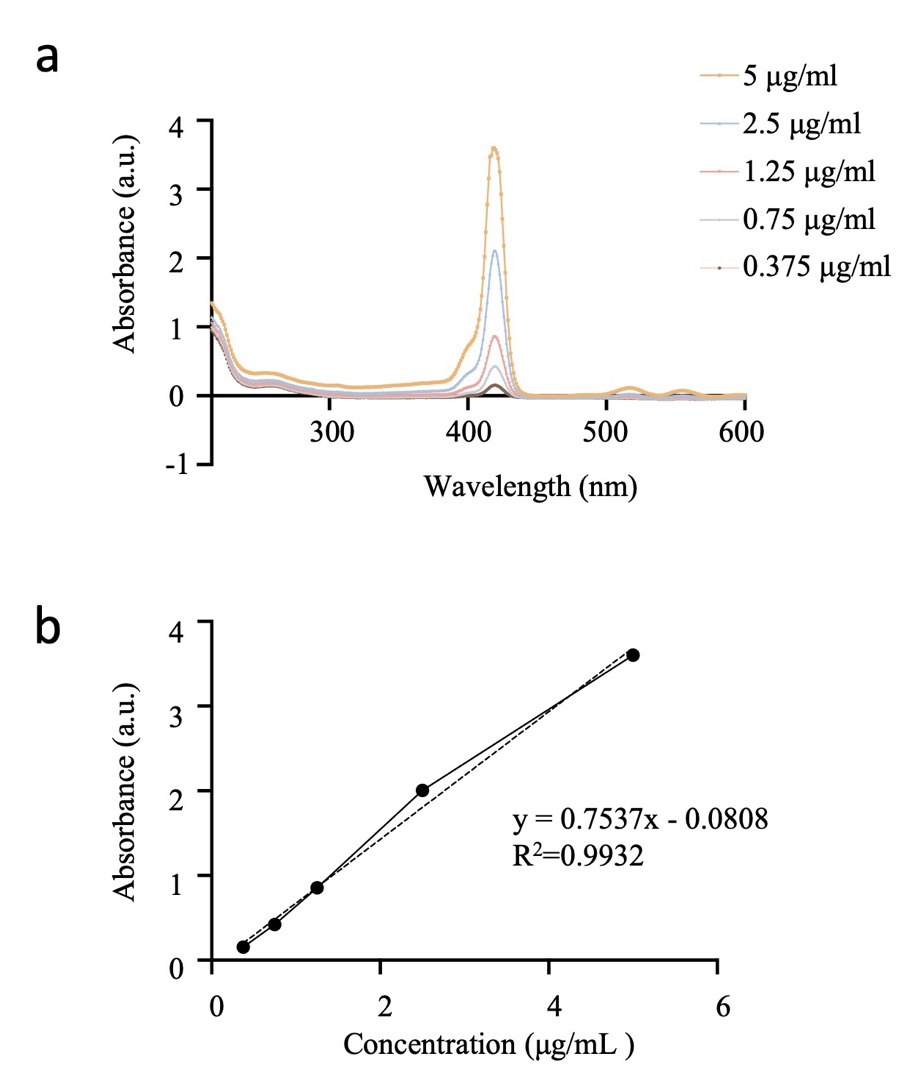


Figure S12: (a) the UV absorption spectra of THPP at different concentrations. (b) The standard curve of THPP.


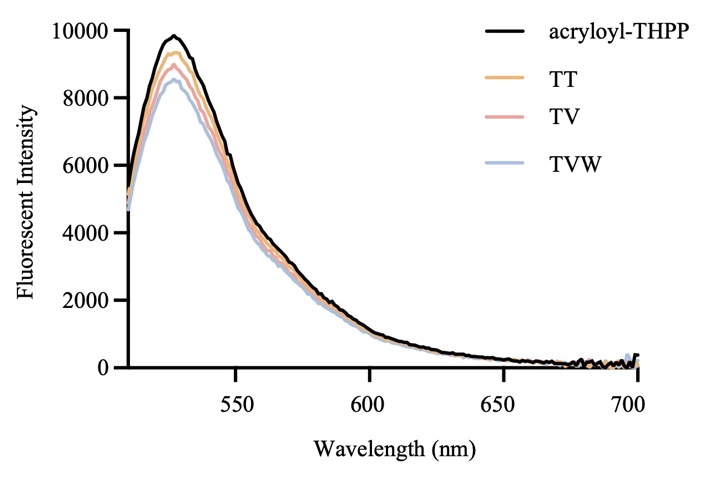


Figure S13: ROS fluorescence intensity generated by acryloyl-THPP, TT, TV, and TVW containing the same amount of acryloyl-THPP


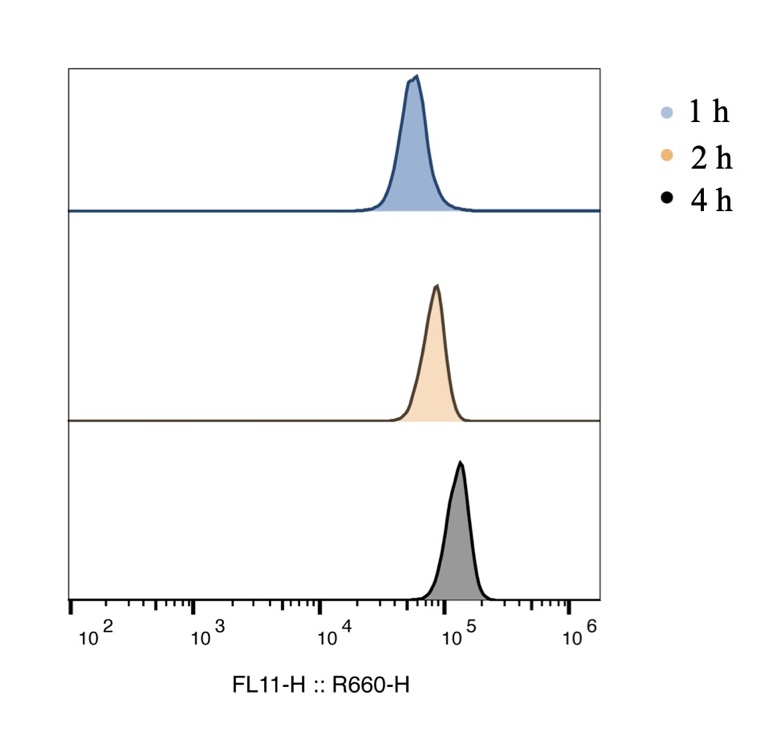


Figure S14: Flow cytometry of Hepa1-6 cells after incubation with TVW for different times.


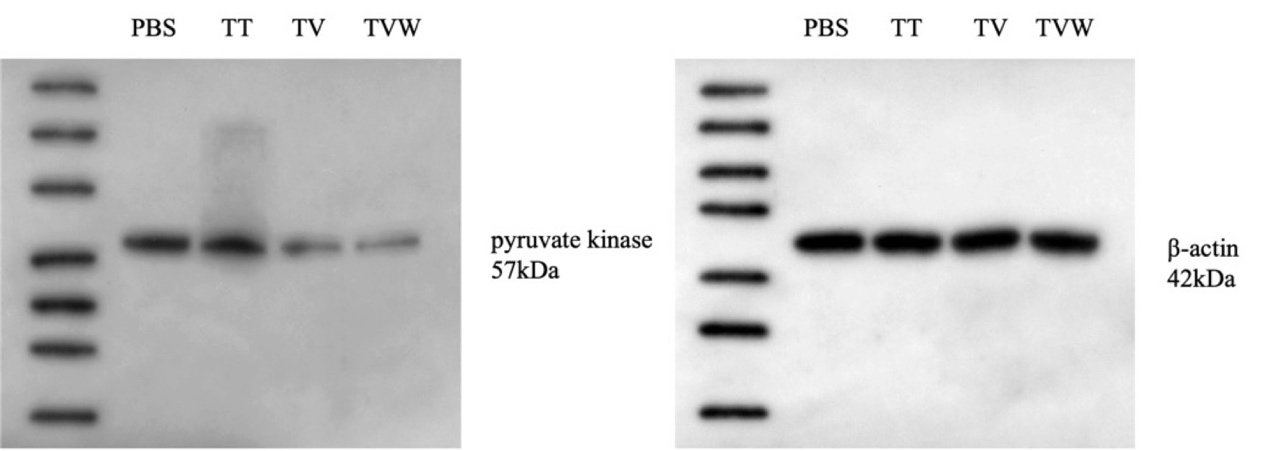


Figure S15: The image of the full gel and blot for Figure 2b.


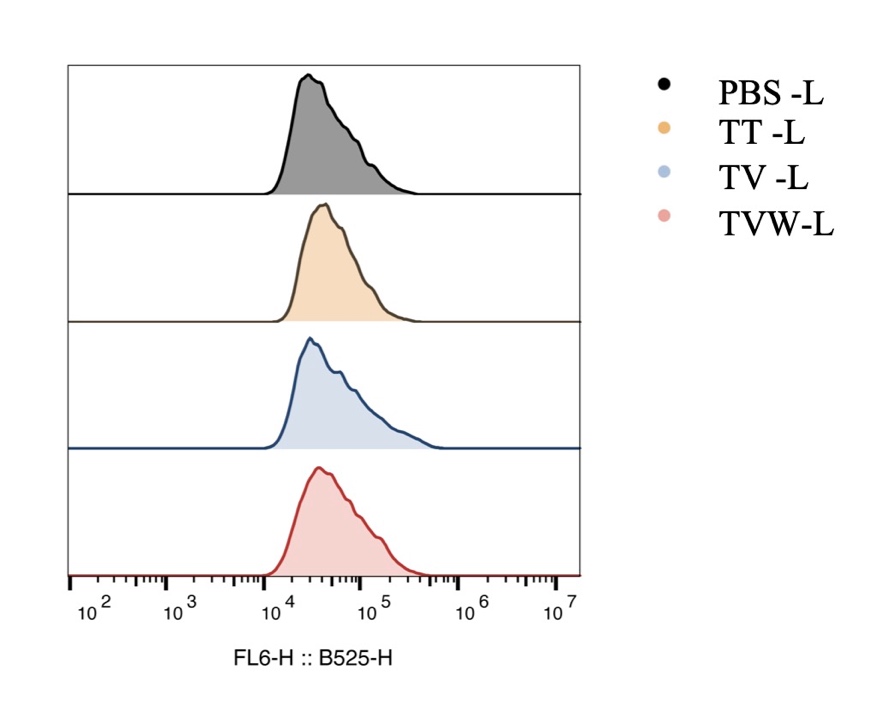


Figure S16: The production of ROS detected by flow cytometry in Hepa1-6 cells.


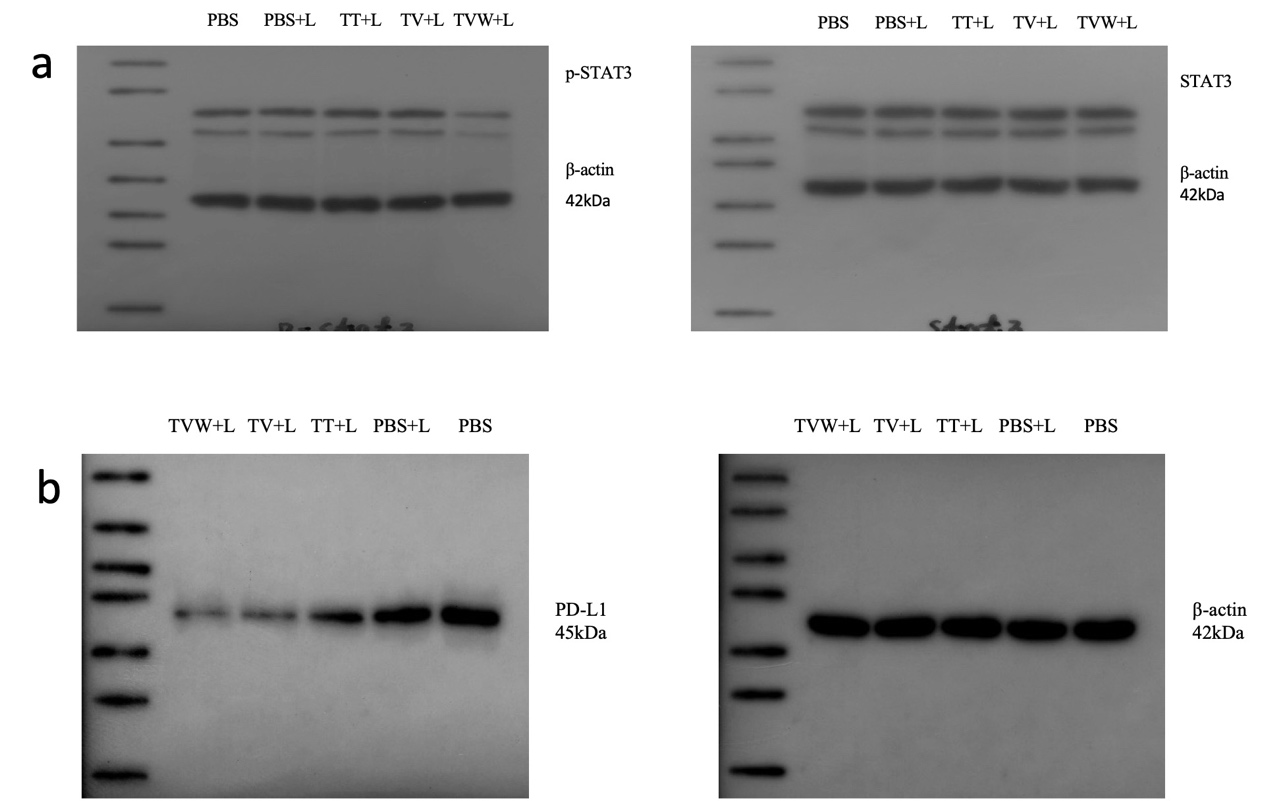


Figure S17: The image of the full gel and blot for Figure 3a.


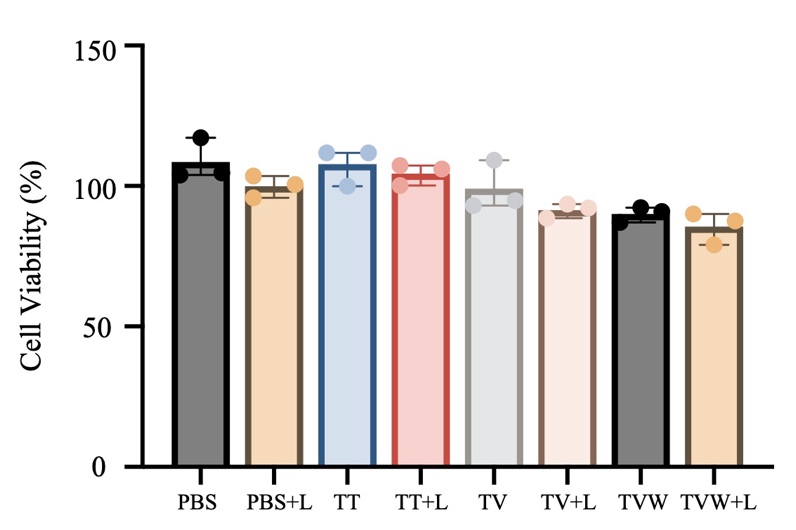


Figure S18: Relative viability of L929 cells after treatment with PBS, PBS+L, TT, TT+L, TV, TV+L, TVW, and TVW+L.


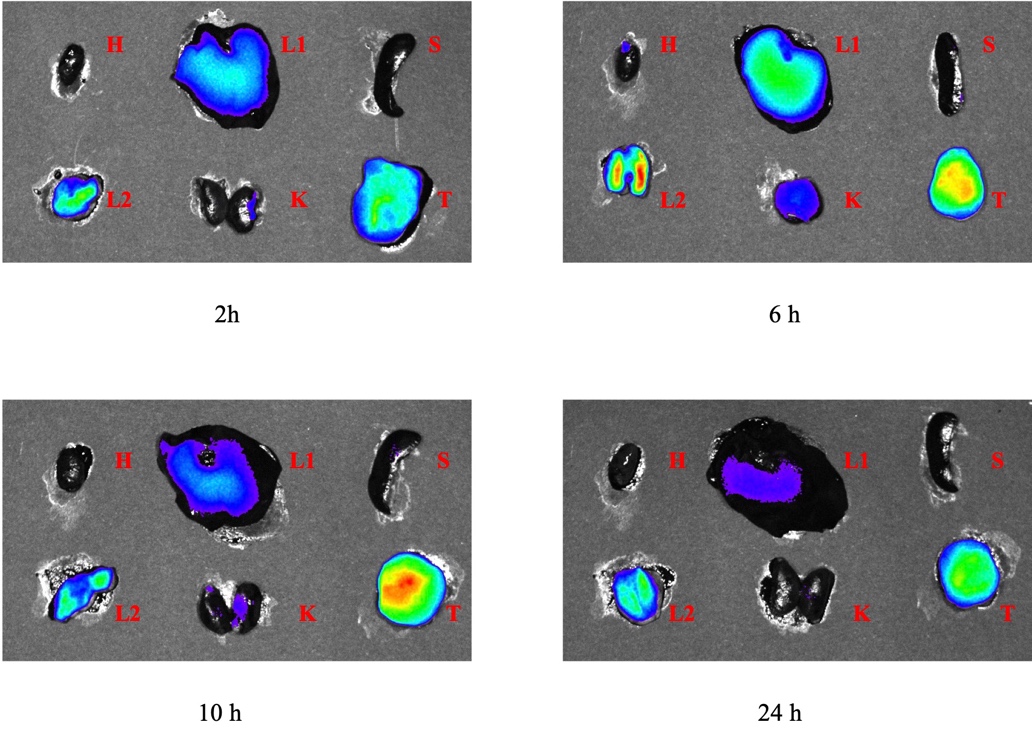


Figure S19: Drug metabolism in the heart, liver, spleen, lungs and kidneys at different time points. (H: heart, L1: liver, S: spleen, L2: lungs, K: kidney and T: tumor)


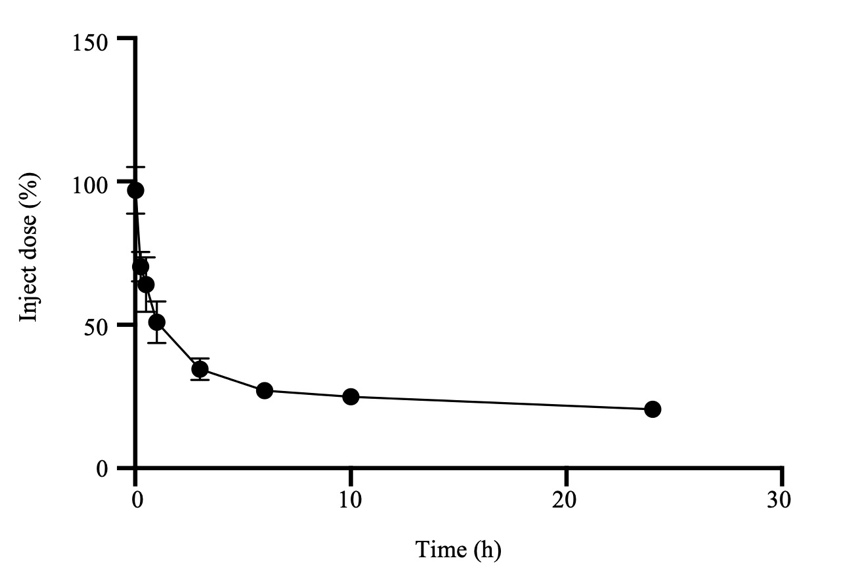


Figure S20: Pharmacokinetics profiles of TVW in the Hepa1-6 tumor-bearing ICR mice after intravenous injection of TVW formulations (n = 3).


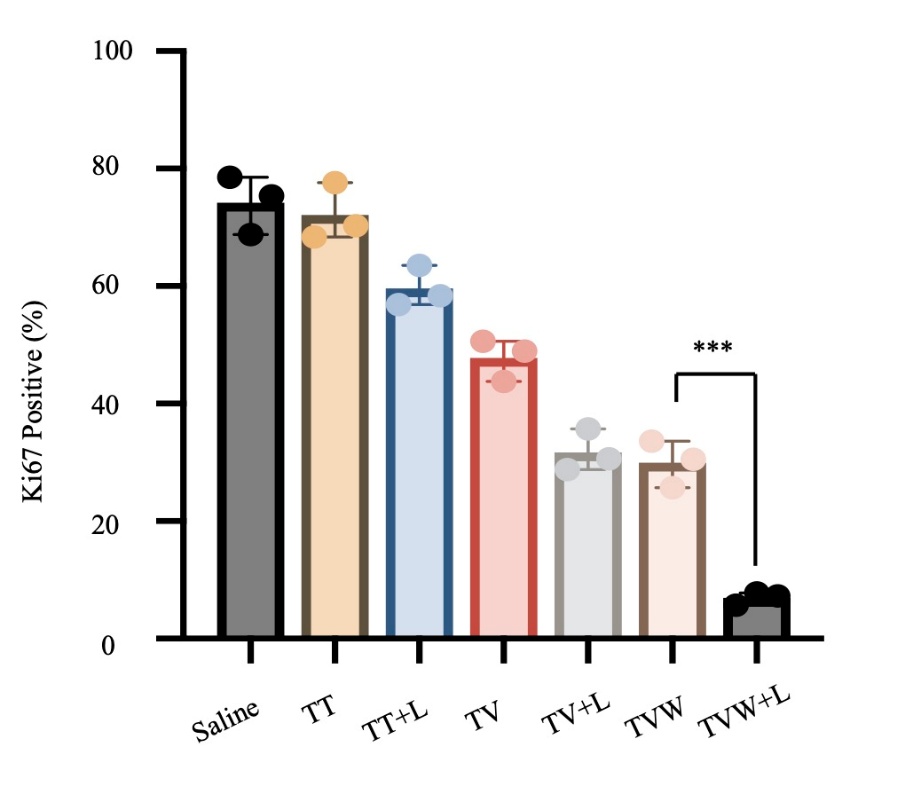


Figure S21: Ki67 statistics after treatment with different groups


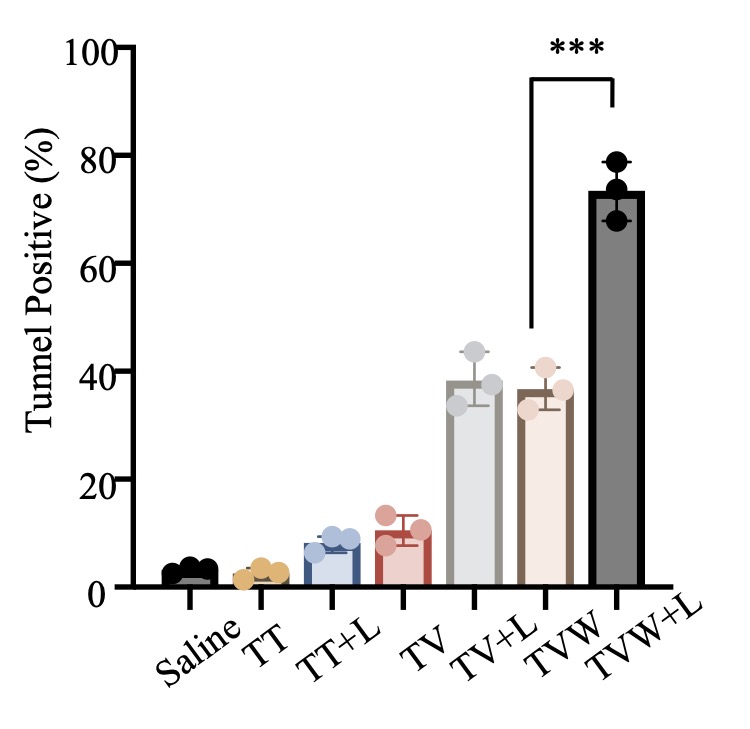


Figure S22: Tunnel statistics after treatment with different groups


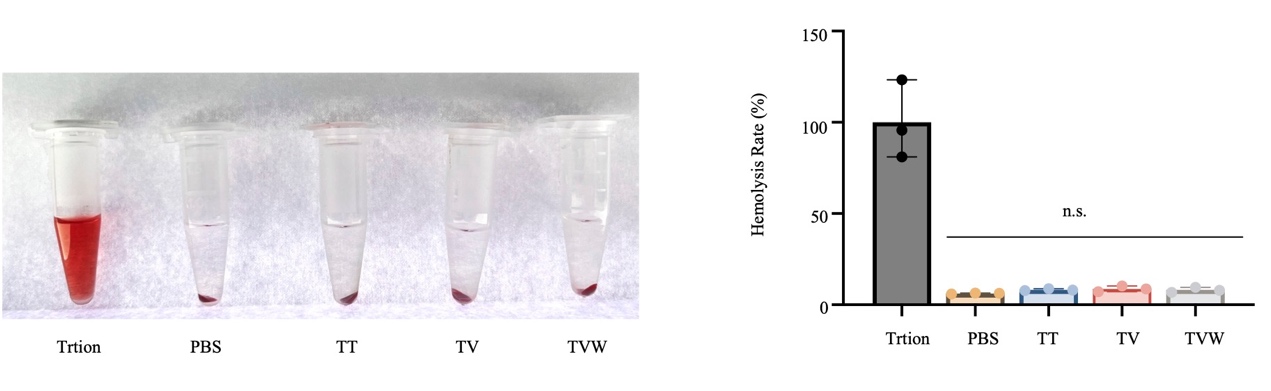


Figure S23: Hemolysis experiments after treatment of different groups.


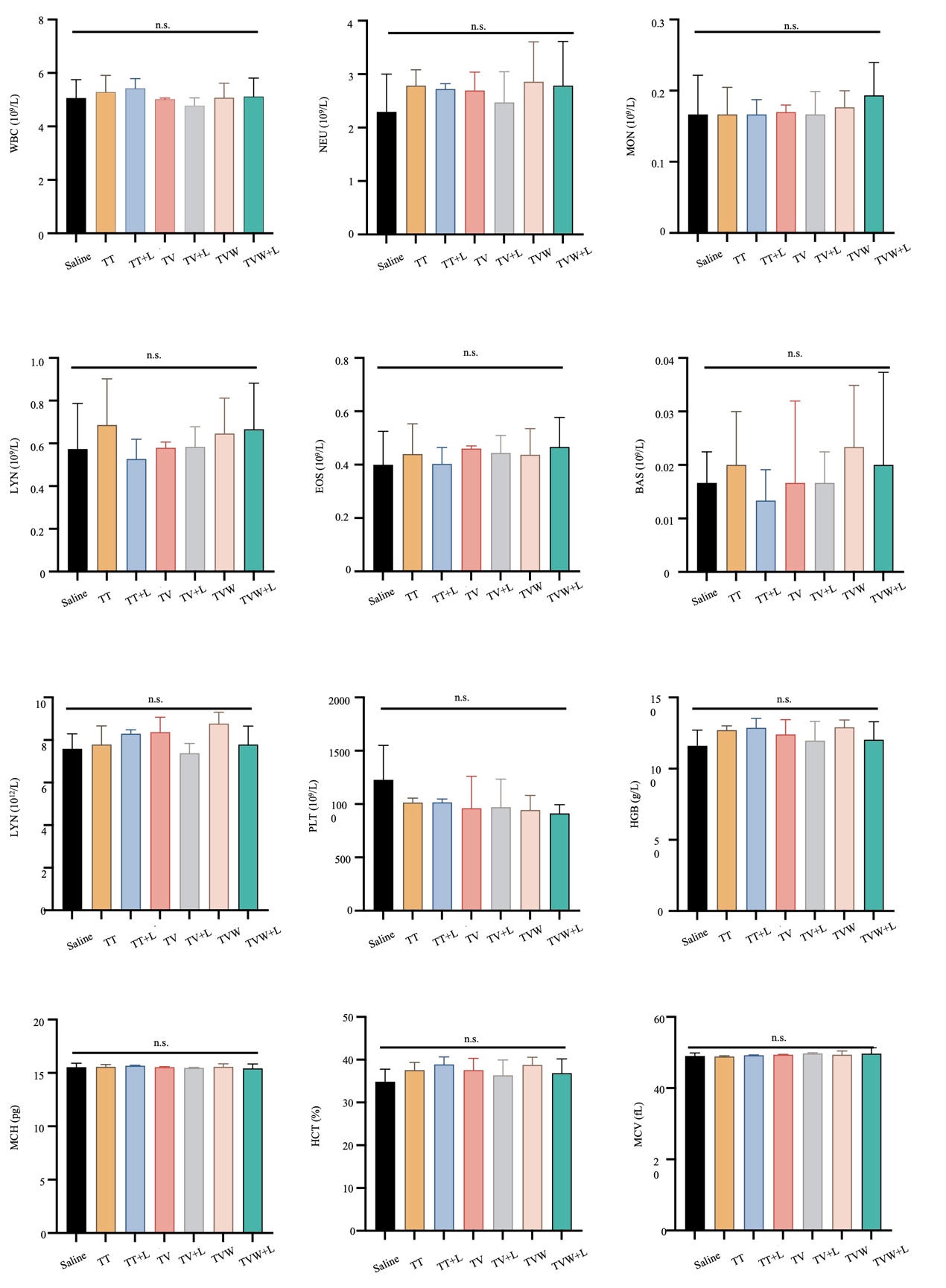


Figure S24: Routine blood tests and blood biochemistry.


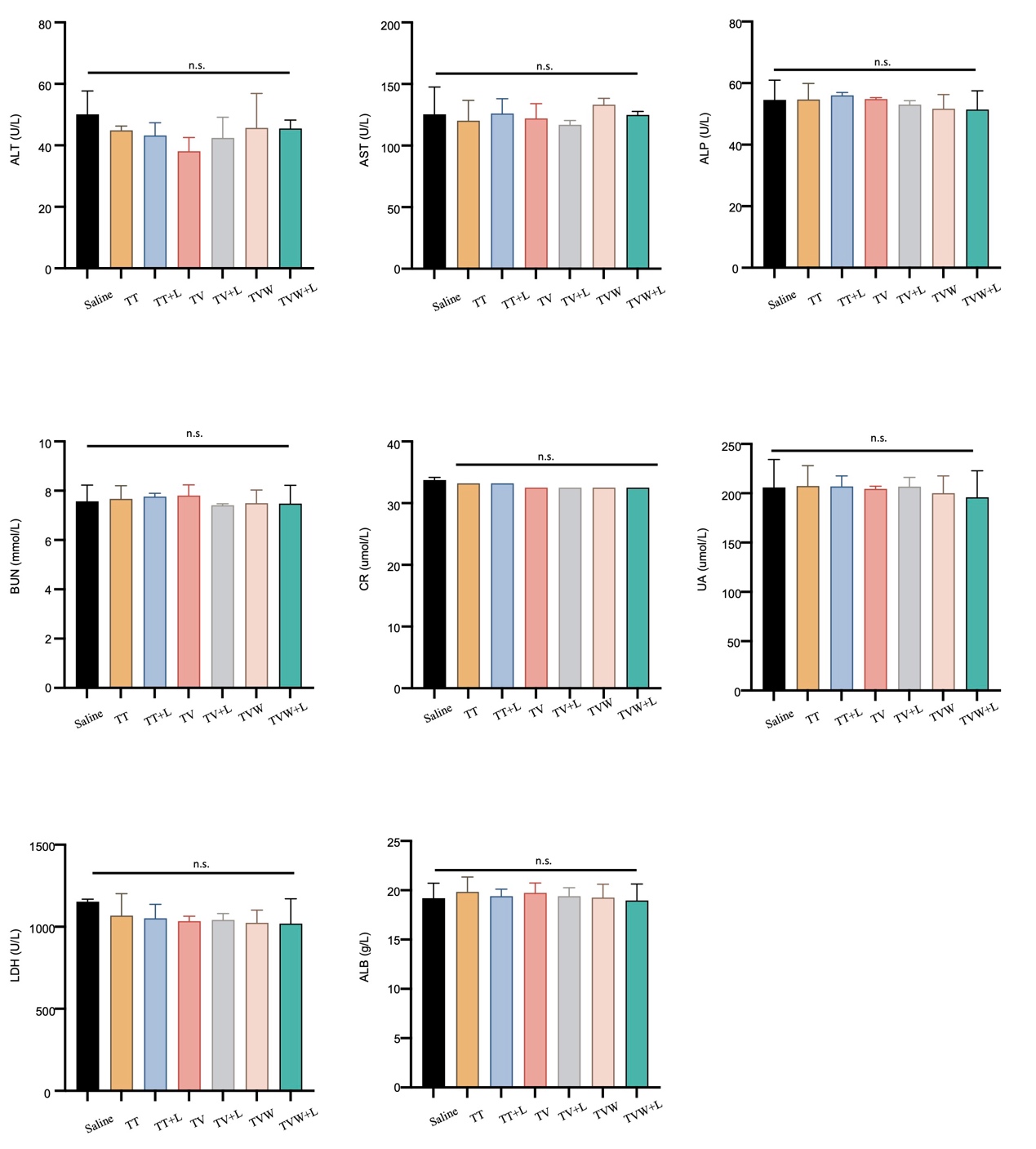


Figure S25: Routine blood tests and blood biochemistry.
